# New sulfurated derivatives of cinnamic acids and rosmaricine as inhibitors of STAT3 and NF-κB transcription factors

**DOI:** 10.1080/14756366.2017.1350658

**Published:** 2017-07-25

**Authors:** Elena Gabriele, Dario Brambilla, Chiara Ricci, Luca Regazzoni, Kyoko Taguchi, Nicola Ferri, Akira Asai, Anna Sparatore

**Affiliations:** a Department of Pharmaceutical Sciences, Università degli Studi di Milano, Milano, Italy;; b Department of Pharmacological and Biomolecular Sciences, Università degli Studi di Milano, Milano, Italy;; c Center for Drug Discovery, Graduate School of Pharmaceutical Sciences, University of Shizuoka, Shizuoka, Japan;; d Department of Pharmaceutical and Pharmacological Sciences, Università degli Studi di Padova, Largo Egidio Meneghetti, Padova, Italy

**Keywords:** Anticancer drug, cytotoxicity, STAT3 inhibitors, NF-κB inhibitors, methanethiosulfonate derivatives

## Abstract

A set of new sulfurated drug hybrids, mainly derived from caffeic and ferulic acids and rosmaricine, has been synthesized and their ability to inhibit both STAT3 and NF-κB transcription factors have been evaluated. Results showed that most of the new hybrid compounds were able to strongly and selectively bind to STAT3, whereas the parent drugs were devoid of this ability at the tested concentrations. Some of them were also able to inhibit the NF-κB transcriptional activity in HCT-116 cell line and inhibited HCT-116 cell proliferation *in vitro* with IC_50_ in micromolar range, thus suggesting a potential anticancer activity. Taken together, our study described the identification of new derivatives with dual STAT3/NF-κB inhibitory activity, which may represent hit compounds for developing multi-target anticancer agents.

## Graphical abstract

**Figure:**
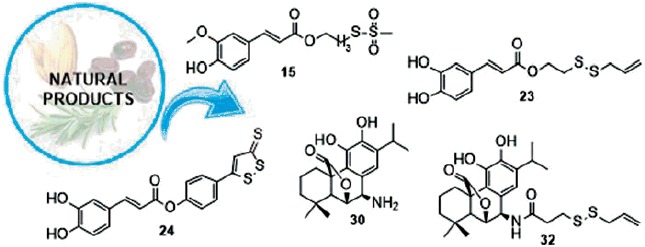

## Introduction

Activation or suppression of STAT signaling play important physiopathological roles in several human diseases (among which cardiovascular, atherosclerosis, rheumatoid arthritis, Alzheimer disease, and particularly cancer).

Research over the last two decades has consolidated the concept that there is a high correlation between tumor development and inflammation: compounds of the inflammatory tumor microenvironment include leukocytes, cytokines, complement components, and are orchestrated by transcription factors, such as Signal Transducer and Activator of Transcription 3 (STAT3) and nuclear factor kappa-light-chain-enhancer of activated B cells (NF-κB)^1^ ^,^ ^2^.

STATs are a family of latent, cytoplasmic transcription factors that are able to regulate cell growth and survival by modulating the expression of specific target genes. STAT3 was found to be constitutively activated by aberrant upstream tyrosine kinase activity in a broad spectrum of cancer cell lines and human tumors, and it is considered a promising target for cancer therapy^3^. All STAT proteins in mammals consist of six domains, as follows: N-domain (ND), coiled-coil, DNA binding, linker, Src homology 2 (SH2), and transcriptional activation domain^4^. Inhibitors exert their pharmacological effects by diverse mechanisms such as blocking abnormally activated upstream kinases such as JAK and Src or directly suppressing the STAT3 phosphorylation^5^. Most of the currently available inhibitors act by preventing STAT3 tyrosine phosphorylation, i.e. they directly bind to the SH2 domain of STAT3 and prevent tyrosine phosphorylation, protein dimerisation, and transcriptional activity^6^ ^,^ ^7^.

STAT3 can directly interact with nuclear factor, NF-κB family member RelA, through acetyltransferase p300-mediated acetylation, trapping it in the nucleus and thereby contributing to constitutive NF-κB activation in tumor-associated hematopoietic cells and various malignancies^7^ ^,^ ^8^.

The NF-κB transcription factor family drives tumor progression and metastasis in many cancers by regulating genes involved in inflammation, cellular survival, and proliferation^9–11^. It is a protein complex able to control DNA transcription and cytokine production and therefore acting with a synergistic effect on cancer development, inflammation, and breast cancer^12^ ^,^ ^13^.

A large number of natural and synthetic compounds, characterised by very different chemical structures, have been described to inhibit STAT3 through several pathways, and the development of novel STAT3 inhibitors is still intensively pursued. Excellent reviews summarise the acquisitions in this field^4^ ^,^ ^14–20^. Particularly relevant for the present study are, the polyphenolic compounds^21^ (as epigallocatechin-3-gallate (EGCG)^22^, resveratrol^23^, curcumin^24^ ^,^ ^25^, butein^26^, ellagic acid^27^, caffeic acid^28^, ferulic acid^29^, carnosic acid, carnosol and related diterpenes^30^, celastrol^31^ and others) and different kinds of sulfurated compounds (α,β-unsaturated sulfones, as stattic^32^; tosyl esters and amides as S3I-201^6^; dithiolethiones^33^, methanethiosulfonates^34^; allicin and diallylpolysulfides^35^; isothiocyanates as sulfuraphane^36^; etc.), which are known to display antiproliferative activity and/or cancer chemopreventive properties, through multiple mechanisms, including STAT3 and/or NF-κB inhibition, either directly or indirectly. The inhibitory potencies versus the relevant proteins are largely variable among the chemotypes and in the different biological settings that are considered.

Agents able to modulate multiple targets can be more efficacious and less prone to produce resistance than drugs that address only a single target; therefore, molecules able to inhibit (one or both) transcription factors by interfering with multiple approaches to their activation pathways could represent useful tools to treat cancer.

In particular, it has been recently demonstrated that dithiolethione derivatives, in addition to the antiangiogenic and tumor suppressor PP2A activation properties^37–39^, inhibited NF-κB transcriptional activity via a covalent reaction, leading to the formation of a disulfide bond with the NF-κB p50 and p65 subunits to inhibit DNA binding in human estrogen receptor–negative breast cancer cells^33^.

Among diallylpolysulfides, diallyl trisulfide (DATS), a constituent of processed garlic, inhibited phosphorylation, dimerisation, and nuclear translocation of STAT3 in prostate cancer cells in culture and *in vivo* ^35^.

**Figure 1.**
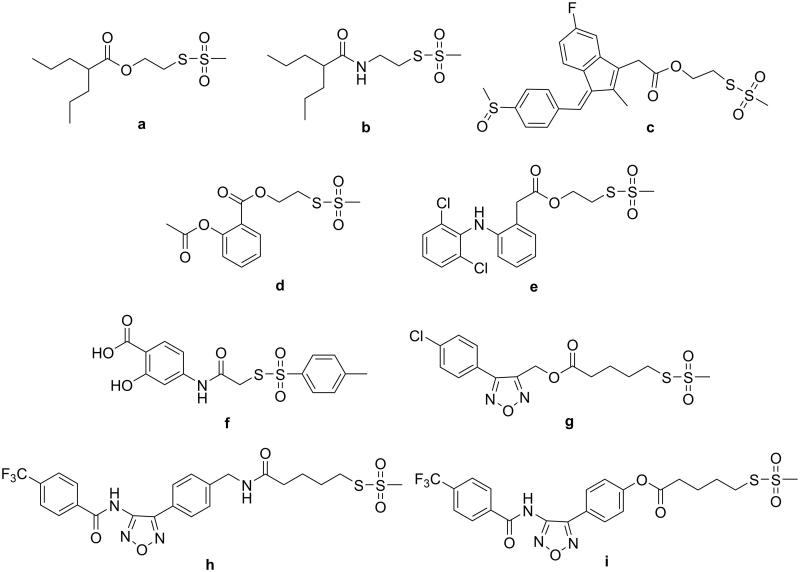
Structures of compounds **a**–**i**.

S-methyl methanethiosulfonate (SMMTS), isolated from cauliflower, is able to inhibit colon tumor incidence when administered to rats during the post-initiation phase of carcinogenesis^34^ and 2-((methylsulfonyl)thio)ethyl 2-propylpentanoate **ACS33** (compound **a**, Figure 1), exhibited *in vitro* good antiproliferative activity in micromolar concentrations on different tumoral cell lines^40^ ^,^ ^41^ and *in vivo* inhibited the growth of PC3 in subcutaneous xenografts^40^.

More recently, we have observed^42^ ^,^ ^43^ that some conjugated molecules (**a**–**i**, Figure 1), incorporating the thiosulfonate function, were able to strongly bind the STAT3-SH2 domain *in vitro* in an AlphaScreen-based assay, with IC_50s_ in the submicromolar-low micromolar range, whereas the parent compounds were devoid of this ability up to the maximum tested concentration (30 μM). Thus, the effected conjugation of the sulfurated and non-sulfurated moieties strongly improved the low or faint affinity to STAT3 of one or both parent compounds.

These compounds exhibited some selectivity for STAT3 inhibition versus STAT1, despite the high degree (78%) of sequence homology between the two STAT proteins.

Compounds **a**, **c**, **d**, and **h** showed a moderate antiproliferative activity (MTT assay) on HCT-116 cells, with IC_50_ from 84 to 135 μM. The other hybrids, as well as all the parent sulfurated and non-sulfurated compounds were inefficacious on HCT-116 cells at concentration up to 200 μM. Since the valuable STAT3 inhibition has been observed in a cell-free assay, the low correspondence between STAT3 inhibition and cytotoxicity could be related to the physicochemical properties of the compounds, which will require optimisation.

On these bases, while attempting to improve the ADME characteristics of the above compounds, we deemed interesting to extend our investigation aimed at the identification of other sulfurated hybrid molecules capable of direct inhibition of STAT3-SH2 domain and, possibly, of improving also the inhibitory activity versus the NF-κB transcription factor that may be simultaneously present in one or both of the parent compounds^44^.

To this purpose, we chose to conjugate several sulfurated moieties (the formerly used and a few others containing different kinds of linking functions) with two phenolic acids (as ferulic and caffeic) and one basic cathecol derivative as rosmaricine, leaving free in all cases the hydroxyl groups to preserve their antioxidant potentiality.

The capability of phenolic acids, particularly caffeic acids and its arylalkyl esters, to interact with STAT3 and NF-κB transcription factors and to exert antiproliferative/antitumoral activity, versus several tumor cell lines in *in vitro* culture and subcutaneous xenografts, is largely documentated^28^ ^,^ ^45–47^.

Phenolic antioxidants, besides acting as scavengers for reactive oxygen intermediates (ROIs, representing signaling molecules to activate NF-κB pathway), may also, for instance, inhibit NF-κB DNA binding. In some settings, caffeic acid inhibited STAT3 with IC_50_s in the range 70–100 μM, while its phenylethyl and phenyl propyl esters displayed IC_50_s in the range 15–30 μM and the improved activity is, at least in part, related to the improved lipophilicity.

On the other hand, rosmaricine is an aminoditerpene, structurally related to carnosic acid, carnosol, and rosmanol, which are endowed with antioxidant, radical scavenger, and antiproliferative activities, through mechanisms that involve, among others, NF-κB and STAT3 inhibition^48–50^. Rosmaricine is obtained from dry leaves of *Rosmarinus officinalis* L. treated with ammonia in the presence of air. It is formed through a complex reaction between some oxidation derivatives of carnosic acid and the ammonia used to liberate the alkaloids supposed to be present in the plant^51^. We have chosen rosmaricine, rather than other phenolic diterpenoids, in the context of a general pharmacological investigation of this unusual molecule, going on since long time^52^ ^,^ ^53^.

In order to evaluate the contribution of the antioxidant activity of the free phenolic groups to the inhibition of transcription factors, some hybrid molecules of non-hydroxylated or variously substituted cinnamic acids were also considered (cinnamic, 3,4-dichlorocinnamic, 3,4-dimethoxycinnamic and 3,4-bis((2-methoxyethoxy)methoxy)cinnamic acids). On the other hand, to evidence the significance of the reactive thiosulfonate function for STAT3 inhibition, the 5-(methylsulfonylthio)pentanoic acid was compared with the structurally close 5-(methylsulfonyl)pentanoic acid, containing the unreactive sulfone group.

**Figure 2.**
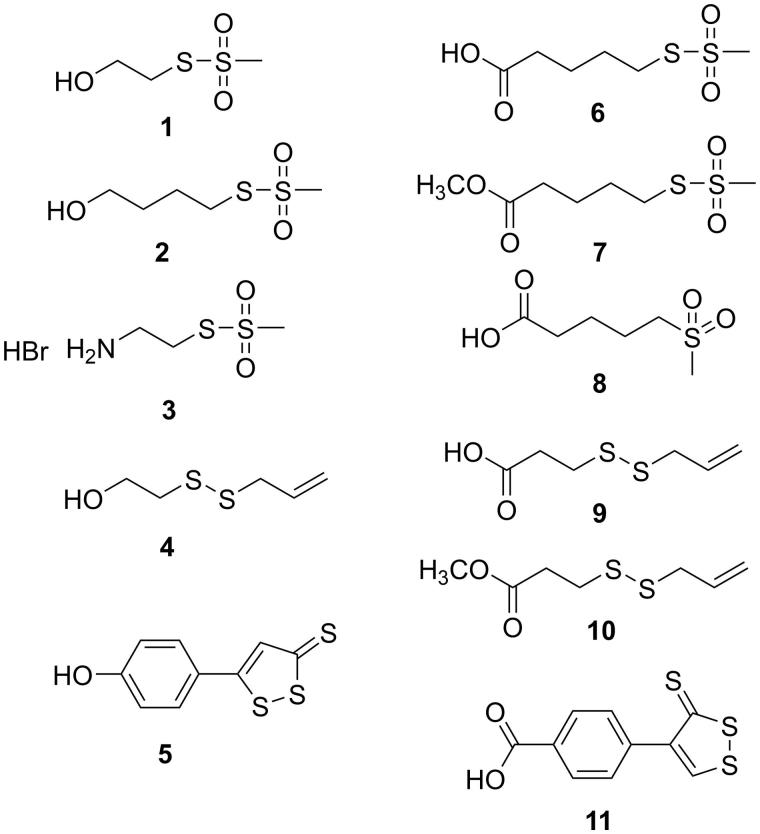
Structures of the investigated sulfurated moieties.

**Figure 3.**
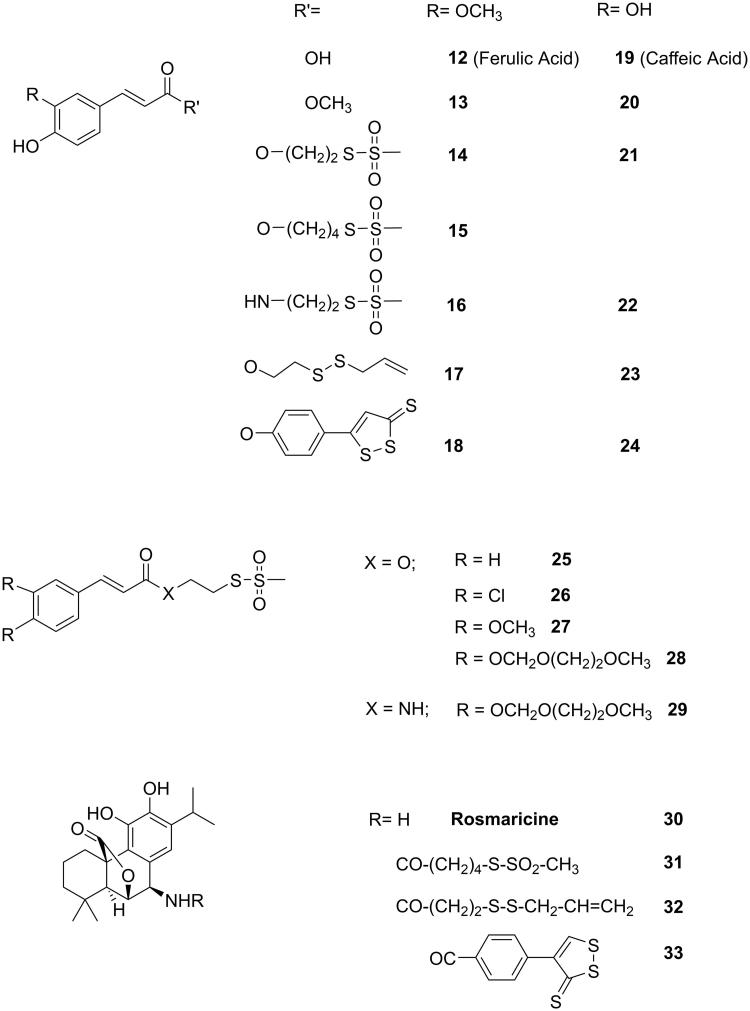
Structures of the investigated derivatives of ferulic, caffeic, and other cinnamic acids and of rosmaricine.

The sulfurated conjugated molecules, together with the parent compounds (Figures 2 and 3) were submitted to the AlphaScreen–based assay^54^ to investigate their ability to interact with STAT3-SH2 domain. Moreover, the cytotoxicity (MTT assay)^55^ of these compounds on HCT-116 cell line (a human colon carcinoma, which express high level of STAT3^56^) was also evaluated. The most active compounds were also submitted to the Luciferase assay^57^ ^,^ ^58^, to measure their ability to inhibit NF-κB promoter and STAT3 reporter activity in HCT-116 and HeLa cells, respectively.

## Materials and methods

### General

All commercially available solvents and reagents were used without further purification, unless otherwise stated; CC = flash column chromatography; melting points (uncorrected) were determined with a Büchi apparatus. ^1^H-NMR and 13C-NMR spectra were recorded with a Bruker DRX Avance 200 MHz (Bruker Italia, Srl, Milano, Italy) or with a Varian 300 MHz Oxford spectrometer (Varian, Palo Alto, CA) equipped with a non-reverse probe at 25° C, in CDCl_3_ or DMSO-d_6_, acetone-d_6_, D_2_O; the chemical shifts were expressed in ppm (*δ*), coupling constants (*J*) in Hertz (Hz). High-resolution mass spectra (HRMS) was performed on a FT-Orbitrap mass spectrometer (Thermo Scientific, Milano, Italy) in positive or negative electrospray ionization (ESI). UPLC-MS analysis: ACQUITY UPLC BEH C18 column, 130 Å, 1.7 μm, 2.1 mm × 50 mm (Waters, Switzerland) and Xevo G2-XS Q-TOF with electrospray ionization source (Waters, Switzerland). MaxEnt 1 software : used to deconvolute the multiple charge states. Microwave reactor: Biotage^®^ initiator classic (Biotage, Uppsala, Sweden). 

The known sulfurated starting compounds **1** ^59^, **3** ^60^, **5** ^61^, **6** ^62^, **9** ^63^, **11** ^63^, and the ferulic and caffeic acid methyl esters **13** ^64^ and **20** ^65^ have been synthesised according to the literature procedures.

### Sulfurated compounds

#### *S*-4-hydroxybutyl methanesulfonothioate (2)

**Scheme 1.**
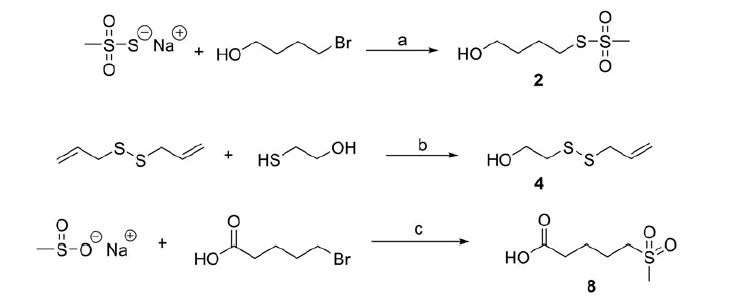
Reagents and conditions: (a) DMF, N_2_, 60 °C, 5 h; (b) DMSO, N_2_, 60 °C, 5 h; (c) dry DMF, 60 °C, 5 h.

Sodium methanethiosulfonate (1.9 g, 14.37 mmol) and 4-bromobutanol (2.0 g, 13.07 mmol) were mixed together in anhydrous DMF (7 ml) under inert atmosphere (Scheme 1). The reaction was stirred at 60 °C for 5 h and was monitored by thin layer chromatography (TLC). After this time, inorganic salts were filtered and the solution was evaporated under reduced pressure. The resulting dark-yellow oil was purified by CC (silica gel; CH_2_Cl_2_/MeOH; in gradient); the product eluted with 0.3% of MeOH. A light brown oil was obtained. Yield: 19%. ^1^H-NMR (300 MHz, DMSO-d_6_): *δ* = 4.45 (br s, 1H, OH collapsed with D_2_O), 3.48 (s, 3H, CH_3_), 3.42 (t, 2H, *J* = 8.7 Hz, CH_2_OH), 3.19 (t, 2H, *J* = 6.9 Hz, SCH_2_), 1.76-1.66 (m, 2H, CH_2_), 1.52–1.43 (m, 2H, CH_2_) ppm. ^13^C-NMR (75 MHz, CDCl_3_): *δ* = 62.0, 50.9, 36.4, 31.3, 26.3. HRMS (ESI) *m/z* Calcd for C_5_H_13_O_3_S_2_ [M + H]^+^: 185.03061; found: 185.03010.

#### 2-(Allyldisulfanyl)ethanol (4)

2-Mercaptoethanol (1.24 g; 0.016 mol) was added to a solution of diallyl disulfide (0.803 g; 0.0055 mol) in DMSO (5 ml) and the mixture was stirred at 50 °C for 5 h under a N_2_ stream. The solution was purified by flash chromatography on silica gel, using cyclohexane/ethyl acetate (68:32) as eluent. A colorless oil was obtained. Yield: 50%. ^1^H-NMR (300 MHz, CDCl_3_): *δ* = 5.92–5.78 (m, 1H), 5.26–5.16 (m, 2H), 3.87 (t, *J* = 5.86 Hz, 2H), 3.34 (d, *J* = 7.33 Hz, 2H), 2.85 (t, *J* = 5.86 Hz, 2H), 1.88 (s, 1H, collapses with D_2_O). ^13^C-NMR (75 MHz, CDCl_3_): *δ* = 133.2, 118.7, 60.2, 42.1, 41.2 ppm. GC/MS (HP 5973; EI): *m/z* 150 [M^+^].

#### Methyl 5-((methylsulfonyl)thio)pentanoate (7)

To a solution of 5-(methylsulfonylthio)pentanoic acid (**22**, 250 mg, 1.18 mmol), DCC (243 mg, 1.18 mmol) and DMAP (13.2 mg, 0.12 mmol) in anhydrous CH_2_Cl_2_ (1.5 ml), MeOH (0.3 ml, 7.40 mmol) was added and the reaction mixture was stirred 3.5 h at r.t., under nitrogen atmosphere. After the filtration of DCU, the solvent was evaporated and the residue was taken up with CH_2_Cl_2._ The organic solution was washed successively with cold 0.5 N HCl, cold 5% (w/w) NaHCO_3_ and finally with iced water and brine and then it was dried with anhydrous Na_2_SO_4_, filtered and evaporated to dryness to yield the pure ester **44** as a pale yellow oil. Yield: 89%. ^1^H-NMR (300 MHz, CDCl_3_): *δ* = 3.67 (s, 3H, OCH_3_), 3.32 (s, 3H, CH_3_), 3.18 (t, 2H, *J* = 7.2 Hz, CH_2_COO), 2.37 (t, 2H, *J* = 7.2 Hz, SCH_2_), 1.83-1.76 (m, 4H, CH_2_) ppm. ^13^C-NMR (75 MHz, CDCl_3_): *δ* = 173.3, 51.6, 50.6, 35.9, 33.1, 28.8, 23.6 ppm. HRMS (ESI) *m/z* Calcd for C_7_H_15_O_4_S_2_ [M + H]^+^: 227.04118; found: 227.04058.

#### 5-(Methylsulfonyl)pentanoic acid (8)

To a solution of sodium methanesulfinate (2 g, 19.03 mmol) in anhydrous *N,N-*dimethylformamide (12 ml), 5-bromovaleric acid (1.73 g, 9.51 mmol) was added. The mixture was stirred at 60 °C for 5 h. The solution was evaporated under reduced pressure and the obtained residue was taken up with iced-water and acidified with a cold solution of 2 M KHSO_4_. The aqueous phase was extracted with ethyl acetate (four times) and the combined organic extracts were washed firstly with a cold solution of 2 M KHSO_4_, then with iced-water and brine. Then, the organic phase was dried with anhydrous Na_2_SO_4_, filtered and evaporated to dryness to obtain a yellow pale oil which crystallised spontaneously in the fridge. The solid was rinsed with a solution of diethyl ether/petroleum ether (1:1) to give white crystals. Yield: 27%; melting point 111.3–112.8 °C. ^1^H-NMR (300 MHz, DMSO-d_6_): *δ* 12.01 (s, 1H, COOH collapsed with D_2_O), 3.13 (t, 2H, *J* = 7.4 Hz, CH_2_), 2.92 (s, 3H, CH_3_), 2.23 (t, 2H, *J* = 7.4 Hz, CH_2_), 1.72–1.53 (m, 4H, CH_2_) ppm. ^13^C-NMR (75 MHz, CD_3_OD): *δ* = 175.4, 53.4, 39.2, 32.7, 23.3, 21.5 ppm. HRMS (ESI) *m/z* Calcd for C_6_H_13_O_4_S [M + H]^+^: 181.05345; found: 181.05287.

#### Methyl 3-(allyldisulfanyl)propanoate (10)

Methanol (0.45 ml, 10.43 mmol) was added to a stirring solution of 3-(allyldisulfanyl)propaonic acid (ACS81, 300 mg, 1.68 mmol), DCC (379.1 mg, 1.84 mmol) and DMAP (17.9 mg, 0.16 mmol) in anhydrous dichloromethane (1.5 ml) under inert atmosphere. The reaction was monitored by thin layer chromatography (eluent phase cyclohexane:ethylacetate/1:1) and was completed within 2 h, stirring at room temperature. After the filtration of DCU, the solvent was evaporated and the residue was taken up with dichloromethane and washed firstly with a cold solution of 0.5 N HCl, then with a cold solution of 5% NaHCO_3_, finally with iced water and brine. The organic layer was dried with anhydrous Na_2_SO_4_, filtered and evaporated to dryness to obtain the final product as a yellow pale oil. Yield: 80%. ^1^H-NMR (300 MHz, CDCl_3_): *δ* 5.91–5.77 (m, 1H, CH = CH_2_), 5.24–5.13 (m, 2H, CH = CH_2_), 3.70 (s, 3H, CH_3_), 3.33 (d, 2H, *J* = 7.5 Hz, CH_2_), 2.93 (t, 2H, *J* = 7.2 Hz, CH_2_), 2.74 (t, 2H, *J* = 7.2 Hz, CH_2_) ppm. 13C-NMR (75 MHz, CD_3_OD): *δ* = 172.5, 133.4, 117.3, 47.1, 41.5, 33.4, 33.3 ppm.

### Ferulic acid derivatives

#### (*E*)-(2-methoxyethoxy)methyl 3-(3-methoxy-4-((2-methoxyethoxy)methoxy)phenyl)acrylate (34)

**Scheme 2.**
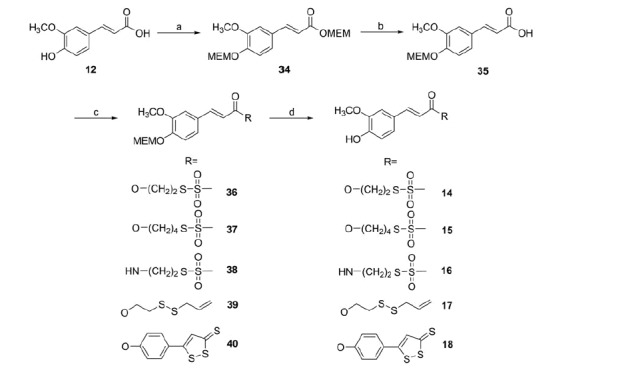
Reagents and conditions: (a) MEM-Cl, DIPEA, anh. CH_2_Cl_2_, 5 h, 0 °C to r.t.; (b) LiOH*H_2_O, THF/H_2_O (2:1), 24 h, r.t.; (c) RH (**1–5**), DCC (EDAC for **38**), DMAP, anh. CH_2_Cl_2_ (CHCl_3_ for **38**), 3–20 h, r.t.; (d) TFA, anh. CH_2_Cl_2_, 4–7 h, r.t.

To a solution of ferulic acid (100 mg, 0.52 mmol) and DIPEA (0.27 ml, 1.03 mmol) in anhydrous CHCl_3_ (2 ml) at 0 °C, 2-methoxyethoxymethyl chloride (0.18 ml, 1.55 mmol) dissolved in anhydrous CHCl_3_ (1 ml) was added dropwise for 5 min (Scheme 2). After 5 min, the ice-bath was removed and the mixture was stirred at r.t. for 5 h under inert atmosphere. After 3 h, 2-methoxyethoxymethyl chloride (0.03 ml, 0.22 mmol) was added in order to speed up the reaction and it was monitored by TLC. After the completion of reaction, the solution was washed with 0.5 N HCl and then with 5% NaHCO_3_. The organic layer was dried with anhydrous Na_2_SO_4_, filtered and evaporated to dryness to obtain a yellow pale oil. Yield: 99%. ^1^H-NMR (300 MHz, DMSO-d_6_): *δ* = 7.64 (d, 1H, *J* = 15.9 Hz, CH = CH), 7.41 (d, 1H, *J* = 1.6 Hz, ArH), 7.23 (d, 1H, *J* = 8.2 Hz, ArH), 7.09 (dd, 1H, *J* = 1.6 and 8.2 Hz, ArH), 6.60 (d, 1H, *J* = 15.9 Hz, CH = CH), 5.36 (s, 2H, OCH_2_O), 5.26 (s, 2H, OCH_2_O), 3.81 (s, 3H, OCH_3_), 3.74–3.70 (m, 4H, CH_2_), 3.48–3.43 (m, 4H, CH_2_), 3.23 (s, 3H, OCH_3_), 3.20 (s, 3H, OCH_3_) ppm. ^13^C-NMR (75 MHz, DMSO-d_6_): *δ* = 166.3, 150.2. 148.7, 145.7, 128.6, 122.9, 116.3, 111.7, 109.9, 93.9, 89.1, 71.4, 71.3, 69.2, 68.0, 58.5, 58.4, 56.1 ppm. HRMS (ESI) *m/z* Calcd for C_18_H_27_O_8_ [M + H]^+^: 371.17059; found: 371.17029.

#### (*E*)-3-(3-methoxy-4-((2-methoxyethoxy)methoxy)phenyl)acrylic acid (35)

(*E*)-(2-Methoxyethoxy)methyl 3-(3-methoxy-4-((2-methoxyethoxy) methoxy) phenyl) acrylate (**34**, 1420 mg, 3.83 mmol) and lithium hydroxide monohydrate (1450 mg, 34.47 mmol) dissolved in 15 ml of THF/H_2_O solution (2:1), were stirred for 24 h at r.t. The reaction was monitored by TLC. After the completion of reaction, the THF was evaporated under reduced pressure. The obtained residue was acidified with a solution of 0.5 N HCl and the aqueous phase was extracted three times with EtOAc. The organic layer was washed with brine and dried with anhydrous Na_2_SO_4_, filtered and evaporated to dryness to obtain a pale pink oil which was crystallised with ethyl ether providing the title compound as a white solid; melting point 78.4–79.8 °C. Yield: 84%. ^1^H-NMR (300 MHz, DMSO-d_6_): *δ* = 12.24 (s, 1H, COOH collapsed with D_2_O), 7.51 (d, 1H, *J* = 15.9 Hz, CH = CH), 7.34 (d, 1H, *J* = 1.6 Hz, ArH), 7.17 (d, 1H, *J* = 8.2 Hz, ArH), 7.08 (dd, 1H, *J* = 1.6 and 8.2 Hz, ArH), 6.46 (d, 1H, *J* = 15.9 Hz, CH = CH), 5.25 (s, 2H, OCH_2_O), 3.80 (s, 3H, OCH_3_), 3.74–3.70 (m, 2H, CH_2_), 3.48–3.43 (m, 2H, CH_2_), 3.20 (s, 3H, OCH_3_) ppm. ^13^C-NMR (75 MHz, CDCl_3_): *δ* = 172.4, 149.7, 148.8, 146.7, 128.3, 122.7, 115.7, 115.5, 110.4, 94.1, 71.4, 67.9, 58.9, 55.8 ppm. HRMS (ESI) *m/z* Calcd for C_14_H_19_O_6_ [M + H]^+^: 383.11816; found: 283.11790.

#### 3-(3-Methoxy-4-((2-methoxyethoxy)methoxy)phenyl)acrylate esters (36, 37, 39, 40) (general method)

To a solution of (*E*)-3-(3-methoxy-4-((2-methoxyethoxy)methoxy)phenyl)acrylic acid (109 mg, 0.53 mmol) and the appropriate ROH (**1, 2**, **3**, **5**; 1 or 1.1 eq) in anhydrous CH_2_Cl_2_ (2–5 ml) or anh. THF (5 ml), DCC (109 mg, 0.53 mmol) and DMAP (7 mg, 0.05 mmol) were added. The reaction mixture was stirred for 5 h at r.t. under inert atmosphere. After the filtration of DCU, the residue was diluted with CH_2_Cl_2_ and washed three times with a cold solution of 0.5 N HCl and then with iced-water and finally with brine. The organic layer was dried with anhydrous Na_2_SO_4_, filtered and the solvent was removed under reduced pressure. The resulting residue was purified by CC (silica gel; CH_2_Cl_2_/MeOH; in gradient as indicated for each compound).

#### (*E*)-2-(methylsulfonylthio)ethyl 3-(3-methoxy-4-((2-methoxyethoxy)methoxy)phenyl)acrylate (36)

CC (silica gel; CH_2_Cl_2_/MeOH; in gradient up to 99:1). The title compound was obtained as a pale yellow oil. Yield: 77%. ^1^H-NMR (300 MHz, CDCl_3_): *δ* = 7.67 (d, 1H, *J* = 15.9 Hz, CH = CH), 7.22 (dd, 1H, *J* = 1.9 and 8.2 Hz, ArH), 7.10 (d, 1H, *J* = 8.2 Hz, ArH), 7.06 (d, 1H, *J* = 1.9 Hz, ArH), 6.32 (d, 1H, *J* = 15.9 Hz, CH = CH), 5.37 (s, 2H, OCH_2_O), 4.52 (t, 2H, *J* = 6.6 Hz, COOCH_2_), 3.92 (s, 3H, OCH_3_), 3.83 (t, 2H, *J* = 3.8, CH_2_), 3.55 (t, 2H, *J* = 3.8, CH_2_), 3.50 (t, 2H, *J* = 6.6 Hz, CH_2_S), 3.40 (s, 3H, CH_3_), 3.37 (s, 3H, OCH_3_) ppm. 13C-NMR (75 MHz, CDCl_3_): *δ* = 167.9, 150.9, 145.5, 140.1, 129.2, 121.0, 116.24, 110.8, 109.9, 95.0, 71.3, 67.7, 62.4, 55.1, 53.3, 39.0, 35.2 ppm.

#### (*E*)-4-(methylsulfonylthio)butyl 3-(3-methoxy-4-((2-methoxyethoxy)methoxy)phenyl)acrylate (37)

CC (silica gel; CH_2_Cl_2_/MeOH; in gradient up to 99.6:0.4). Yellow oil. Yield: 70%. ^1^H-NMR (300 MHz, DMSO-d_6_): *δ* = 7.61 (d, 1H, *J* = 15.6 Hz, CH = CH), 7.39 (d, 1H, *J* = 1.5 Hz, ArH), 7.21 (dd, 1H, *J* = 1.5 and 8.1 Hz, ArH), 7.09 (d, 1H, *J* = 8.1 Hz, ArH), 6.59 (d, 1H, *J* = 15.6 Hz, CH = CH), 5.25 (s, 2H, OCH_2_O), 4.19–4.15 (m, 2H, COOCH_2_), 3.79 (s, 3H, OCH_3_), 3.73–3.68 (m, 2H, CH_2_), 3.51 (s, 3H, CH_3_), 3.48–3.42 (m, 2H, CH_2_), 3.30–3.22 (m, 3H, CH_2_S), 3.21 (s, 2H, OCH_3_), 1.80–1.72 (m, 4H, CH_2_) ppm. 13C-NMR (75 MHz, CDCl_3_): *δ* = 167.0, 149.7, 148.6, 144.8, 128.6, 122.3, 115.9, 115.8, 110.3, 94.1, 71.5, 67.9, 63.4, 59.0, 55.9, 50.7, 35.9, 27.6, 26.3 ppm.

#### (*E*)-2-(allyldisulfanyl)ethyl 3-(3-methoxy-4-((2-methoxyethoxy)methoxy)phenyl)acrylate (39)

CC (silica gel; CH_2_Cl_2_/MeOH; in gradient up to 99.9:0.1). The title compound was obtained as an old-rose oil. Yield: 80%. ^1^H-NMR (300 MHz, CDCl_3_): *δ* = 7.68 (d, 1H, *J* = 15.9 Hz, CH = CH), 7.31 (d, 1H, *J* = 1.3 Hz, ArH), 7.22 (d, 1H, *J* = 8.2 Hz, ArH), 7.12 (dd, 1H, *J* = 1.3 and 8.2 Hz, ArH), 6.38 (d, 1H, *J* = 15.9 Hz, CH = CH), 5.96-5.90 (m, 1H, CH = CH_2_), 5.41 (s, 2H, OCH_2_O), 5.29–5.17 (m, 2H, CH = CH_2_), 4.51–4.43 (m, 2H, CH_2_), 3.93 (s, 3H, OCH_3_), 3.88–3.84 (m, 2H, CH_2_), 3.59–3.56 (m, 2H, CH_2_), 3.39 (s, 3H, OCH_3_), 3.38–3.35 (m, 2H, CH_2_), 3.09–2.97 (m, 2H, CH_2_) ppm. 13C-NMR (75 MHz, CDCl_3_): *δ* = 166.8, 149.7, 148.6, 145.1, 133.2, 128.6, 122.4, 118.7, 115.8, 115.7, 110.2, 94.1, 71.4, 67.9, 62.4, 58.9, 55.8, 42.3, 37.3 ppm.

#### (*E*)-4-(3-thioxo-3*H*-1,2-dithiol-5-yl)phenyl 3-(3-methoxy-4-((2-methoxyethoxy)methoxy)phenyl) acrylate (40)

The solid residue was crystallised with a solution of CH_2_Cl_2_/MeOH (3:0.5) to give the title compound as an orange solid. Yield: 68%; melting point 105.7–108.2 °C. ^1^H-NMR (300 MHz, DMSO-d_6_): *δ* = 7.99 (d, 2H, *J* = 7.9 Hz, ArH), 7.84 (s, 1H, CH = C), 7.82 (d, 1H, *J* = 15.9 Hz, CH = CH), 7.50 (d, 1H, *J* = 1.6 Hz, ArH), 7.40 (d, 2H, *J* = 7.9 Hz, ArH), 7.33 (d, 1H, *J* = 8.2 Hz, ArH), 7.13 (dd, 1H, *J* = 1.6 and 8.2 Hz, ArH), 6.84 (d, 1H, *J* = 15.9 Hz, CH = CH), 5.29 (s, 2H, OCH_2_O), 3.83 (s, 3H, OCH_3_), 3.73 (t, 2H, *J*= 5.8 Hz, CH_2_), 3.45 (t, 2H, *J*= 5.8 Hz, CH_2_), 3.21 (s, 3H, OCH_3_) ppm. 13C-NMR (75 MHz, DMSO-d_6_): *δ* = 216.1, 173.4, 165.5, 154.2, 150.6, 148.7, 146.6, 136.4, 135.6, 126.1, 124.5, 123.6, 116.3, 113.2, 111.9, 94.5, 71.4, 68.1, 66.4, 58.5 ppm.

#### (*E*)-*S*-2-(3-(3-methoxy-4-((2-methoxyethoxy)methoxy)phenyl)acrylamido)ethyl methanesulfonothioate (38)

*S*-2-aminoethyl methanesulfonothioate hydrobromide **(3**, 230 mg, 0.97 mmol) and DIPEA (0.17 ml, 0.97 mmol) were mixed together in anhydrous CHCl_3_ (8 ml) and (*E*)-3-(3-methoxy-4-((2-methoxyethoxy)methoxy) phenyl)acrylic acid (250 mg, 0.89 mmol), EDAC (254 mg, 1.34 mmol) and DMAP (11 mg, 0.09 mmol) were added at r.t. The reaction mixture was stirred for 4 h under inert atmosphere and it was monitored by TLC. The obtained residue was poured into a separation funnel, firstly washed with a cold solution of 0.5 N HCl, then with cold water and finally with brine. The organic layer was dried with anhydrous Na_2_SO_4_, filtered and the solvent was stripped off to obtain the title compound as a yellow pale oil. Yield: 96%. ^1^H-NMR (300 MHz, DMSO-d_6_): *δ* = 8.85 (s, 1H, NH collapsed with D_2_O), 7.38 (d, 1H, *J* = 15.6 Hz, CH = CH), 7.19 (d, 1H, *J* = 1.8 Hz, ArH), 7.11 (dd, 1H, *J* = 1.8 and 8.5 Hz, ArH), 7.09 (d, 1H, *J* = 8.5 Hz, ArH), 6.51 (d, 1H, *J* = 15.6 Hz, CH = CH), 5.23 (s, 2H, OCH_2_O), 3.79 (s, 3H, OCH_3_), 3.73–3.70 (m, 2H, NHCH_2_), 3.54 (s, 3H, CH_3_), 3.51 (t, 2H, *J* = 6 Hz, CH_2_), 3.46–3.43 (m, 2H, CH_2_S), 3.31 (t, 2H, *J* = 6 Hz, CH_2_), 3.20 (s, 3H, OCH_3_) ppm. 13C-NMR (75 MHz, CD_3_OD): *δ* = 167.7, 150.2, 148.0, 140.7, 129.4, 121.2, 118.4, 116.5, 110.7, 94.0, 71.4, 67.6, 57.6, 55.0, 49.3, 38.8, 35.2 ppm.

#### 3-(4-Hydroxy-3-methoxyphenyl)acrylate esters (14, 15, 17, 18) and amide (16) (general method)

To a solution of the appropriate 3-(3-methoxy-4-((2-methoxyethoxy)methoxy)phenyl)acrylate derivative (**32–36**) (0.38 mmol) in anhydrous CH_2_Cl_2_ (2 ml), trifluoroacetic acid (0.16 ml, 2.12 mmol) was added and the mixture was stirred at r.t. for 5-7 h. The reaction was monitored by TLC and after its completion, the solvent and the trifluoroacetic acid were evaporated *in vacuo*. The obtained residue was diluted with CH_2_Cl_2_, washed twice with a cold solution of NaHCO_3_ at 5% (w/w) and then with iced-brine. The organic layer was dried with anhydrous Na_2_SO_4_, filtered and evaporated to dryness to obtain a residue that was purified by CC (silica gel; eluent as indicated for each compound).

#### (*E*)-2-(methylsulfonylthio)ethyl 3-(4-hydroxy-3-methoxyphenyl)acrylate (14)

CC (CH_2_Cl_2_; isocratic): a colorless oil was obtained. Yield: 64%. ^1^H-NMR (300 MHz, DMSO-d_6_): *δ* = 9.64 (br s, 1H, OH collapsed with D_2_O), 7.58 (d, 1H, *J* = 15.9 Hz, CH = CH), 7.32 (d, 1H, *J* = 1.9 Hz, ArH), 7.12 (dd, 1H, *J* = 1.9 and 8.2 Hz, ArH), 6.78 (d, 1H, *J =* 8.2 Hz, ArH), 6.48 (d, 1H, *J* = 15.9 Hz, CH = CH), 4.41 (t, 2H, *J* = 6.1 Hz, COOCH_2_), 3.80 (s, 3H, OCH_3_), 3.58 (s, 3H, CH_3_), 3.53 (t, 2H, *J* = 6.1 Hz, CH_2_S) ppm. 13C-NMR (75 MHz, CDCl_3_): *δ* = 166.7, 148.3, 146.8, 146.1, 126.6, 123.2, 144.8, 114.2, 109.5, 62.2, 55.9, 50.9, 35.1 ppm. HRMS (ESI) *m/z* Calcd for C_13_H_17_O_6_S_2_ [M + H]^+^: 333.04665; found: 333.04606.

#### (*E*)-4-(methylsulfonylthio)butyl 3-(4-hydroxy-3-methoxyphenyl)acrylate (15)

CC (CH_2_Cl_2_/MeOH; in gradient up to 99.5:0.5). Light-gray oil. Yield: 64%. ^1^H-NMR (300 MHz, DMSO-d_6_): *δ* = 9.60 (br s, 1H, OH collapsed with D_2_O), 7.53 (d, 1H, *J* = 15.7 Hz, CH = CH), 7.30 (d, 1H, *J* = 1.4 Hz, ArH), 7.10 (dd, 1H, *J_1_*=1.4 and 8.6 Hz, ArH), 6.78 (d, 1H, *J* = 8.6 Hz, ArH), 6.45 (d, 1H, *J* = 15.7 Hz, CH = CH), 4.14 (t, 2H, *J* = 6.15 Hz, COOCH_2_), 3.79 (s, 3H, OCH_3_), 3.51 (s, 3H, CH_3_), 3.30–3.22 (m, 3H, CH_2_S), 1.80–1.72 (m, 4H, CH_2_) ppm. 13C-NMR (75 MHz, CDCl_3_): *δ* = 167.1, 148.0, 146.8, 145.1, 126.8, 123.1, 115.1, 114.7, 109.4, 63.3, 55.9, 50.7, 35.9, 27.6, 26.3 ppm. HRMS (ESI) *m/z* Calcd for C_15_H_21_O_6_S_2_ [M + H]^+^: 361.07795; found: 361.07734.

#### (*E*)-S-2-(3-(4-hydroxy-3-methoxyphenyl)acrylamido)ethyl methanesulfonothioate (16)

CC (CH_2_Cl_2_/MeOH; in gradient up to 99.5:0.5). Light-gray oil. Yield: 50%. ^1^H-NMR (300 MHz, DMSO-d_6_): *δ* = 9.46 (s, 1H, OH collapsed with D_2_O), 9.29 (s, 1H, NH collapsed with D_2_O), 7.34 (d, 1H, *J* = 15.6 Hz, CH = CH), 7.12 (d, 1H, *J* = 1.8 Hz, ArH), 6.99 (dd, 1H, *J* = 1.8 and 7.9 Hz, ArH), 6.78 (d, 1H, *J* = 7.9 Hz, ArH), 6.42 (d, 1H, *J* = 15.6 Hz, CH = CH), 3.79 (s, 3H, OCH_3_), 3.56–3.50 (m, 2H, NHCH_2_), 3.54 (s, 3H, CH_3_), 3.32–3.30 (m, 2H, CH_2_S) ppm. 13C-NMR (75 MHz, DMSO-d_6_): *δ* = 166.4, 149.1, 148.5, 140.2, 126.9, 122.3, 119.0, 116.3, 111.5, 56.2, 50.9, 39.0, 36.1. HRMS (ESI) *m/z* Calcd for C_13_H_18_NO_5_S_2_ [M + H]^+^: 332.06264; found: 332.06205.

#### (*E*)-2-(allyldisulfanyl)ethyl 3-(4-hydroxy-3-methoxyphenyl)acrylate (17)

CC (CH_2_Cl_2_/MeOH; in gradient 99.9:0.1). Colorless oil. Yield: 35%. ^1^H-NMR (300 MHz, DMSO-d_6_): *δ* = 9.62 (br s, 1H, OH collapsed with D_2_O), 7.59 (d, 1H, *J* = 15.9 Hz, CH = CH), 7.37 (d, 1H, *J* = 1.1 Hz, ArH), 7.11 (d, 1H, *J* = 8.2 Hz, ArH), 7.81 (dd, 1H, *J* = 1.1 and 8.2 Hz, ArH), 6.49 (d, 1H, *J* = 15.9 Hz, CH = CH), 5.91–5.73 (m, 1H, CH = CH_2_), 5.29–5.08 (m, 2H, CH = CH_2_), 4.48–4.31 (m, 2H, COOCH_2_), 3.82 (s, 3H, OCH_3_), 3.42–3.38 (m, 2H, CH_2_S), 3.07–2.96 (m, 2H, SCH_2_) ppm. 13C-NMR (75 MHz, CDCl_3_): *δ* = 166.9, 148.1, 146.7, 145.4, 133.2, 126.8, 123.2, 118.7, 114.9, 114.7, 109.3, 62.1, 55.9, 42.3, 37.3 ppm. HRMS (ESI) *m/z* Calcd for C_15_H_17_O_4_S_2_ [M–H]^–^: 325.05683; found: 325.0549.

#### (*E*)-4-(3-thioxo-3*H*-1,2-dithiol-5-yl)phenyl 3-(4-hydroxy-3-methoxyphenyl)acrylate (18)

After the completion of reaction, the solution was filtered and the resulting solid residue was rinsed with a solution of ethyl ether/CH_2_Cl_2_ (4:1) for two times to provide orange crystals. Yield: 72%. Melting point 204.5–206.8 °C. ^1^H-NMR (300 MHz, DMSO-d_6_): *δ* = 9.75 (br s, 1H, OH collapsed with D_2_O), 7.99 (d, 2H, *J* = 8.7 Hz, ArH), 7.80 (d, 1H, *J* = 15.9 Hz, CH = CH), 7.44 (s, 1H, ArH), 7.39 (m, 3H, ArH), 7.22 (dd, 1H, *J* = 1.6 and 8.2 Hz, ArH), 6.28 (d, 1H, *J* = 8.2 Hz, ArH), 6.74 (d, 1H, *J* = 15.9 Hz, CH = CH), 3.83 (s, 3H, OCH_3_) ppm. 13C-NMR (75 MHz, DMSO-d_6_): *δ* = 216.1, 173.4, 165.5, 154.3, 150.7, 148.7, 148.4, 136.4, 129.3, 126.0, 124.5, 123.8, 116.2, 113.5, 112.2, 56.4. HRMS (ESI) *m/z* Calcd for C_19_H_15_O_4_S_3_ [M + H]^+^: 403.01325; found: 403.01282.

### Caffeic acid derivatives

#### (*E*)-methyl 3-(3,4-bis((2-methoxyethoxy)methoxy)phenyl)acrylate (41)

**Scheme 3.**
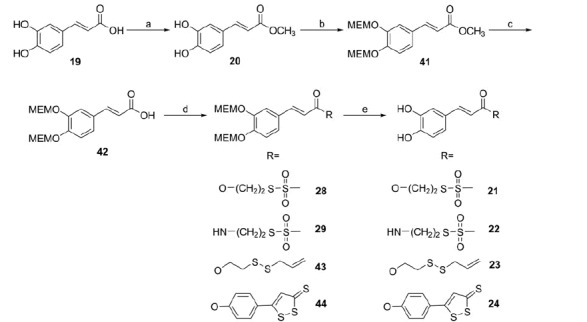
Reagents and conditions: (a) H_2_SO_4_ (cat.), MeOH, reflux, 2 h; (b) NaH, MEM-Cl, anh. THF, 0 °C, 5 h; (c) 5 N NaOH, THF/MeOH (4:1), 40 °C, 3 h; (d) RH (**1**, **3–5**), DCC or EDAC (for **29**), DMAP, anh. THF or CH_2_Cl_2_ or CHCl_3_, 4–20 h, r.t.; (e) TFA, anh. CH_2_Cl_2_ or CHCl_3_, 5–8 h, r.t.

To a solution of 60% sodium hydride (w/w) in mineral oil (375 mg, 9.37 mmol) dissolved in anhydrous THF (12 ml) at 0 °C under nitrogen, (*E*)-methyl 3-(3,4-dihydroxyphenyl)acrylate (**20**, 500 mg, 2.58 mmol) diluted in anhydrous THF (6 ml) was added dropwise (Scheme 3). After 10 min, 2-methoxyethoxymethyl chloride (1.30 g, 10.32 mmol) in anhydrous THF (5 ml) was added slowly and the mixture was stirred at 0 °C for 5 h under inert atmosphere. After its completion, a saturated solution of NH_4_Cl was added and the aqueous phase was extracted three times with CH_2_Cl_2_. The organic layer was dried with anhydrous Na_2_SO_4_, filtered and the solvent was evaporated under reduced pressure to provide a residue that was purified by CC (silica gel; EtOAc/cyclohexane in ratio 7:3; isocratic). The fractions containing the purified product were gathered up and the title compound was obtained as a yellow oil. Yield: 75%. ^1^H-NMR (300 MHz, DMSO-d_6_): *δ* = 7.56 (d, 1H, *J* = 15.9 Hz, CH = CH), 7.48 (d, 1H, *J* = 1.2 Hz, ArH), 7.31 (d, 1H, *J* = 8.2 Hz, ArH), 7.12 (dd, 1H, *J* = 1.2 and 8.2 Hz, ArH), 6.49 (d, 1H, *J* = 15.9 Hz, CH = CH), 5.27 (s, 4H, OCH_2_O), 3.85–3.62 (m, 7H, CH_3_ and CH_2_), 3.52–3.37 (m, 4H, CH_2_), 3.20 (s, 6H, CH_3_) ppm. 13C-NMR (75 MHz, CDCl_3_): *δ* = 167.5, 149.1, 147.2, 144.4, 128.8, 123.5, 116.3, 116.1, 115.9, 94.5, 94.1, 71.4, 71.3, 67.9, 67.8, 59.0, 58.9, 51.6 ppm. HRMS (ESI) *m/z* Calcd for C_18_H_26_O_8_Na [M + Na]^+^: 393.15254; found: 393.15189.

#### (*E*)-3-(3,4-bis((2-methoxyethoxy)methoxy)phenyl)acrylic acid (42)

(*E*)-methyl 3-(3,4-bis((2-methoxyethoxy)methoxy)phenyl)acrylate (1.33 g, 3.59 mmol) dissolved in a mixture of THF/MeOH in ratio 4:1 (16 ml), was treated with a solution of 5 N NaOH (4.3 ml). The resulting mixture was stirred at 40 °C for 3 h. After the completion of reaction, THF and MeOH were evaporated to dryness and the basic aqueous phase was washed with EtOAc in order to remove the unreacted ester. The aqueous mixture was acidified with a solution of 6 N HCl and was extracted three times with EtOAc. The organic layer was dried with anhydrous Na_2_SO_4_, filtered and evaporated *in vacuo* to obtain a yellow oil, which was crystallised with diethyl ether: (*E*)-3-(3,4-bis((2-methoxyethoxy)methoxy)phenyl)acrylic acid was obtained as white solid. Melting point 56.6–58.3 °C. Yield: 98%. ^1^H-NMR (300 MHz, DMSO-d_6_): *δ* = 12.08 (s, 1H, COOH collapsed with D_2_O), 7.48 (d, 1H, *J* = 15.9 Hz, CH = CH), 7.44 (d, 1H, *J* = 1.5 Hz, ArH), 7.27 (dd, 1H, *J* = 1.5 and 8.2 Hz, ArH), 7.12 (d, 1H, *J* = 8.2 Hz, ArH), 6.38 (d, 1H, *J* = 15.9 Hz, CH = CH), 5.26 (s, 4H, OCH_2_O), 3.80–3.65 (m, 4H, CH_2_), 3.55–3.38 (m, 4H, CH_2_), 3.21 (s, 6H, CH_3_) ppm. 13C-NMR (75 MHz, CDCl_3_): *δ* = 171.9, 149.4, 147.2, 146.2, 128.5, 123.9, 116.1, 116.0, 115.9, 94.4, 93.9, 71.4, 71.3, 67.9, 67.8, 59.0, 58.9 ppm. HRMS (ESI) *m/z* Calcd for C_17_H_25_O_8_ [M + H]^+^: 357.15494; found: 357.15489.

#### 3-(3,4-bis((2-Methoxyethoxy)methoxy)phenyl)acrylate esters (28, 43, 44) (general method)

(*E*)-3-(3,4-bis((2-methoxyethoxy)methoxy)phenyl)acrylic acid (355 mg, 1 mmol) and the appropriate ROH **(1, 3**, **5**; 1 mmol) were mixed together in anhydrous CH_2_Cl_2_ or THF (4 ml), and DCC (206 mg, 1 mmol) and a catalytic amount of DMAP were added at r.t. The reaction mixture was stirred for 5–20 h under inert atmosphere and it was monitored by TLC. After the filtration of DCU, the solvent was evaporated under reduced pressure and the residue was dissolved in ethyl acetate and washed three times first with a cold solution of 0.5 N HCl, then with a cold solution of 5% (w/w) NaHCO_3_ and finally with cold brine. The organic layer was dried with anhydrous Na_2_SO_4_, filtered and the solvent was removed under reduced pressure. The resulting residue was purified by CC (silica gel; CH_2_Cl_2_/MeOH or EtOAc/cyclohexane in gradient as indicated for each compound).

#### (*E*)-2-(methylsulfonylthio)ethyl 3-(3,4-bis((2-methoxyethoxy)methoxy)phenyl)acrylate (28)

CC (CH_2_Cl_2_/MeOH; in gradient up to 98.4:1.6). Yellow oil. Yield: 53%. ^1^HNMR (300 MHz, DMSO-d_6_): *δ* = 7.59 (d, 1H, *J* = 15.9 Hz, CH = CH), 7.50 (d, 1H, *J* = 1.2 Hz, ArH), 7.32 (dd, 1H, *J* = 1.2 and 8.2 Hz, ArH), 7.13 (d, 1H, *J* = 8.2 Hz, ArH), 6.50 (d, 1H, *J* = 15.9 Hz, CH = CH), 5.27 (s, 4H, OCH_2_O), 4.42 (t, 2H, *J* = 6 Hz, CH_2_), 3.80–3.65 (m, 4H, CH_2_), 3.57 (s, 3H, CH_3_), 3.53 (t, 2H, *J* = 6 Hz, CH_2_), 3.50-3.38 (m, 4H, CH_2_), 3.21 (s, 6H, CH_3_) ppm. 13C-NMR (75 MHz, CDCl_3_): *δ* = 166.6, 149.4, 147.3, 145.6, 128.5, 123.7, 116.1, 115.8, 115.4, 94.5, 94.1, 77.2, 71.5, 68.0, 67.9, 62.3, 59.1, 50.9, 50.9, 35.2 ppm. HRMS (ESI) *m/z* Calcd for C_18_H_26_O_10_S_2_Na [M + Na]^+^: 517.11781; found: 517.11682.

#### (*E*)-2-(allyldisulfanyl)ethyl 3-(3,4-bis((2-methoxyethoxy)methoxy)phenyl)acrylate (43) and N-acylurea by-product (45)

(a) The raw yellow oil initially obtained was partially crystallised in freezer; after dilution with ether and filtration, white crystals have been obtained, which resulted to be the N-acylurea (**45**). (b) The ethereal mothers, evaporated to dryness were purified through CC (CH_2_Cl_2_/MeOH; in gradient up to 99:1) to achieve compound (**43**).

#### (*E*)-3-(3,4-bis((2-methoxyethoxy)methoxy)phenyl)-*N*-cyclohexyl-*N*-(cyclohexylcarbamoyl) acrylamide (45)

^1^H-NMR (300 MHz, CDCl_3_): *δ* = 7.58 (d, 1H, *J* = 15.6 Hz, CH = CH), 7.34 (d, 1H, *J* = 1.4 Hz, ArH), 7.21 (d, 1H, *J* = 8.2 Hz), 7.10 (dd, 1H, *J* = 1.4 and 8.2 Hz, ArH), 7.05 (br s, 1H, NH collapsed with D_2_O), 6.61 (d, 1H, *J* = 15.6 Hz, CH = CH), 5.33 (s, 2H, CH_2_), 5.30 (s, 2H, CH_2_) 4.10–4.09 (m, 1H, CH), 3.87–3.83 (m, 4H, CH_2_), 3.75–3.73 (m, 1H, CH), 3.57–3.36 (s, 6H, OCH_3_), 1.99–1.58 (m, 10H, CH), 1.41–1.14 (m, 10H, CH) ppm. ^13^C-NMR (75 MHz, CDCl_3_): *δ* = 167.1, 154.1, 149.0, 147.3, 143.1, 129.2, 122.9, 118.06, 116.3, 116.2, 94.6, 94.1, 77.0, 71.5, 71.4, 67.9, 59.0, 56.2, 49.8, 32.8, 30.9, 26.2, 26.2, 25.4, 25.3, 24.66 ppm. HRMS (ESI) *m/z* Calcd for C_30_H_47_N_2_O_8_ [M + H]^+^: 563.33324; found: 563.33222.

#### (*E*)-2-(Allyldisulfanyl)ethyl 3-(3,4-bis((2-methoxyethoxy)methoxy)phenyl)acrylate (43)

Yellow oil. Yield: 40%. ^1^H-NMR (300 MHz, DMSO-d_6_): *δ* = 7.56 (d, 1H, *J* = 15.6 Hz, CH = CH), 7.48 (d, 1H, *J* = 1.2 Hz, ArH), 7.30 (dd, 1H, *J* = 1.2 mand 8.2 Hz, ArH), 7.11 (d, 1H, *J* = 8.2 Hz, ArH), 6.49 (d, 1H, *J* = 15.6 Hz, CH = CH), 5.90–5.70 (m, 1H, CH = CH_2_), 5.40–5.00 (m, 6H, OCH_2_O and CH = CH_2_), 4.35 (d, 2H, *J* = 6.3 Hz, CH_2_), 3.85–3.60 (m, 4H, CH_2_), 3.52–3.35 (m, 6H, CH_2_), 3.20 (s, 6H, OCH_3_), 3.01 (d, 2H, *J* = 6.3 Hz, CH_2_) ppm. ^13^C-NMR (75 MHz, CDCl_3_): *δ* = 166.8, 149.2, 147.2, 144.8, 133.2, 128.7, 123.6, 118.8, 116.1, 115.9, 109.9, 94.5, 94.0, 71.4, 71.3, 68.0, 67.9, 62.4, 59.1, 59.0, 42.3, 37.2 ppm.

#### (*E*)-4-(3-thioxo-3H-1,2-dithiol-5-yl)phenyl 3-(3,4-bis((2-methoxyethoxy)methoxy)phenyl) acrylate (44)

CC (EtOAc/cyclohexane; in gradient up to 37:63). A red oil was obtained which crystallized spontaneously in the fridge during the night. The solid was rinsed with petroleum ether to give orange crystals. Yield: 60%. Melting point 81.4–84.0 °C. ^1^H-NMR (300 MHz, DMSO-d_6_): *δ* = 7.98 (d, 2H, *J* = 8.8 Hz, ArH), 7.84–7.76 (m, 2H, CH = CH and ArH), 7.59 (d, 1H, *J* = 1.2 Hz, ArH), 7.47–7.32 (m, 3H, ArH), 7.17 (d, 1H, *J* = 8.8, ArH), 6.76 (d, 1H, *J* = 15.9 Hz, CH = CH), 5.30 (s, 4H, OCH_2_O), 3.78–3.70 (m, 4H, CH_2_), 3.50–3.41 (m, 4H, CH_2_), 3.21 (s, 6H, OCH_3_) ppm. ^13^C-NMR (75 MHz, DMSO-d_6_): *δ* = 215.9, 173.2, 165.1, 154.0, 150.1, 147.3, 147.2, 136.2, 129.1, 128.2, 124.9, 123.6, 117.2, 116.7, 115.3, 94.3, 93.9, 71.4, 71.3, 68.1, 68.0, 58.5, 68.4 ppm.

#### (*E*)-*S*-2-(3-(3,4-bis((2-methoxyethoxy)methoxy)phenyl)acrylamido)ethyl methanesulfonothioate (29)

*S*-2-aminoethyl methanesulfonothioate hydrobromide (**3,** 101 mg, 0.65 mmol) and DIPEA (0.11 ml, 0.65 mmol) were mixed together in anhydrous CHCl_3_ (4 ml) and (*E*)-3-(3,4-bis((2-methoxyethoxy)methoxy)phenyl)acrylic acid (212 mg, 0.59 mmol), EDAC (107 mg, 0.89 mmol) and DMAP (7.3 mg, 0.06 mmol) were added at r.t. The reaction mixture was stirred for 4 h under argon and it was monitored by TLC. The obtained residue was poured into a separation funnel, firstly washed with a cold solution of 0.5 N HCl, then with a cold solution of 5% (w/w), NaHCO_3_ and finally with cold brine. The organic layer was dried with anhydrous Na_2_SO_4_, filtered and the solvent was removed under reduced pressure to obtain a yellow oil, which crystallised spontaneously in the fridge during the night. The solid was rinsed with ethyl ether to give ash gray crystals. Yield: 80%. Melting point 62.3–64.6 °C. ^1^H-NMR (300 MHz, DMSO-d_6_): *δ* = 8.38 (s, 1H, NH collapsed with D_2_O), 7.38–7.30 (m, 2H, ArH and CH = CH), 7.17 (m, 2H, ArH), 6.46 (d, 1H, *J* = 15.6 Hz, CH = CH), 5.26 (s, 4H, OCH_2_O), 3.78–3.69 (m, 4H, CH_2_), 3.60–3.39 (m, 9H, CH_3_ and CH_2_), 3.32 (t, 2H, *J* = 6.3 Hz, CH_2_S), 3.20 (s, 6H, OCH_3_) ppm. ^13^C-NMR (75 MHz, CD_3_OD): *δ* = 167.6, 149.0, 147.4, 140.4, 129.3, 122.9, 118.6, 116.7, 116.0, 94.3, 94.0, 71.4, 71.3, 67.7, 67.6, 57.7, 57.6, 49.3, 38.7, 35.1 ppm. HRMS (ESI) *m/z* Calcd for C_18_H_28_NO_9_S_2_ [M + H]^+^: 494.15185; found: 494.15112.

#### 3-(3,4-Dihydroxyphenyl)acrylate esters (21, 23, 24) and amide (22) (general method)

To a solution of the appropriate 3-(3,4-dihydroxyphenyl)acrylate derivative (**28, 29, 43, 44**; 0.42 mmol) in anhydrous CH_2_Cl_2_ or CHCl_3_ (2 ml), trifluoroacetic acid (0.32 ml, 4.21 mmol) was added and the reaction mixture was stirred, under argon, at r.t. for 5–8 h. After the completion of reaction, the solvent and the trifluoroacetic acid were evaporated to dryness. The residue was washed with the solvents indicated for each compounds or purified by CC (silica, eluent as indicated).

#### (*E*)-2-(methylsulfonylthio)ethyl 3-(3,4-dihydroxyphenyl)acrylate (21)

Solid residue washed with CH_2_Cl_2_/petroleum ether (1:1) and dried *in vacuo* to afford the title compound as a white solid. Yield: 64%. Melting point 133.1–134.8 °C. ^1^H-NMR (300 MHz, DMSO-d_6_): *δ* = 9.61 (br s, 1H, OH collapsed with D_2_O), 9.13 (br s, 1H, OH collapsed with D_2_O), 7.50 (d, 1H, *J* = 15.9 Hz, CH = CH), 7.04–7.00 (m, 2H, ArH), 6.47 (d, 1H, *J* = 8.9 Hz, ArH), 6.25 (d, 1H, *J* = 15.9 Hz, CH = CH), 4.39 (t, 2H, *J* = 6.3 Hz, CH_2_), 3.56 (s, 3H, CH_3_), 3.51 (t, 2H, *J* = 6.3 Hz, CH_2_) ppm. ^13^C-NMR (75 MHz, d_6_-DMSO): *δ* = 166.9, 149.3, 146.5, 146.2, 126.0, 122.2, 116.4, 115.6, 113.9, 62.8, 50.8, 35.1. HRMS (ESI) *m/z* Calcd for C_12_H_15_O_6_S_2_ [M + H]^+^: 319.03100; found: 319.03052.

#### (*E*)-*S*-2-(3-(3,4-dihydroxyphenyl)acrylamido)ethyl methanesulfonothioate (22)

The obtained residue was purified by CC (silica gel; CH_2_Cl_2_/MeOH; in gradient up to 99:1). The title compound was obtained as a tan oil. Yield: 9%. ^1^H-NMR (300 MHz, DMSO-d_6_): *δ* = 8.25 (s, 2H, –OH collapsed with D_2_O), 7.54 (s, 1H, NH collapsed with D_2_O), 7.42 (d, 1H, *J* = 15.6 Hz, CH = CH), 7.08 (d, 1H, *J* = 1.1 Hz, ArH), 6.95 (dd, 1H, *J* = 1.1 and 8.2 Hz, ArH), 6.84 (d, 1H, *J* = 8.2 Hz, ArH), 6.44 (d, 1H, *J* = 15.6 Hz, CH = CH), 3.78–3.60 (m, 2H, NHCH_2_), 3.49 (s, 3H, CH_3_), 3.41 (t, 2H, *J* = 6.3 Hz, CH_2_S) ppm. ^13^C-NMR (75 MHz, DMSO-d_6_): *δ* = 166.2, 147.9, 145.9, 140.1, 126.6, 120.9, 118.2, 116.1, 114.2, 50.6, 38.8, 35.8 ppm. HRMS (ESI) *m/z* Calcd for C_12_H_16_NO_5_S_2_ [M + H]^+^: 318.04699; found: 318.04643.

#### (*E*)-2-(allyldisulfanyl)ethyl 3-(3,4-dihydroxyphenyl)acrylate (23)

The obtained residue was taken up with CH_2_Cl_2_ and washed twice with iced-brine. The organic layer was dried with anhydrous Na_2_SO_4_, filtered and evaporated to dryness to obtain a residue that was purified by preparative TLC (silica; eluent mixture: CH_2_Cl_2_/MeOH 10:0.4). The title compound was obtained as a green-gray oil. Yield: 42%. ^1^H-NMR (300 MHz, acetone-d_6_): *δ* = 8.45 (s, 1H, OH collapsed with D_2_O), 8.17 (s, 1H, OH collapsed with D_2_O), 7.55 (d, 1H, *J* = 15.2 Hz, CH = CH), 7.16 (s, 1H, ArH), 7.05 (dd, 1H, *J* = 1.8 and 7.9 Hz, ArH), 6.86 (d, 1H, *J* = 7.9 Hz, ArH), 6.28 (d, 1H, *J* = 15.2 Hz, CH = CH), 5.92–5.82 (m, 1H, CH = CH_2_), 5.25–5.11 (m, 2H, CH = CH_2_), 4.40 (t, 1H, *J* = 6.6 Hz, CH_2_)*, 4.28 (t, 1H, *J* = 6.6 Hz, CH_2_)*, 3.42 (d, 1H, *J* = 7.1 Hz, CH_2_)*, 3.26 (d, 1H, *J* = 7.1 Hz, CH_2_)*, 3.03 (t, 1H, *J* = 6.3 Hz, CH_2_)*, 2.75 (t, 1H, *J* = 6.3 Hz, CH_2_)* ppm. ^13^C-NMR (75 MHz, acetone-d_6_): *δ* = 166.3, 147.9, 145.4, 145.2, 133.5, 126.6, 121.7, 118.0, 115.5, 114.4, 124.3, 61.9, 41.6, 36.9 ppm. * The ^1^H NMR showed split signals for the enantiotropic hydrogens. HRMS (ESI) *m/z* Calcd for C_14_H_15_O_4_S_2_ [M–H]^–^: 311.04118; found: 311.0395.

#### (*E*)-4-(3-thioxo-3*H*-1,2-dithiol-5-yl)phenyl 3-(3,4-dihydroxyphenyl)acrylate (24)

The residue treated with crystallised CH_2_Cl_2_. The orange crystals were filtered and washed with ethyl ether/MeOH (9:1) to provide the title compound as an orange solid. Yield: 54%. Melting point 199.5–201.5 °C. ^1^H-NMR (300 MHz, DMSO-d_6_): *δ* = 9.72 (br s, 1H, OH collapsed with D_2_O), 9.18 (br s, 1H, OH collapsed with D_2_O), 7.97 (d, 2H, *J* = 8.5 Hz, ArH), 7.82 (s, 1H, ArH), 7.71 (d, 1H, *J* = 15.9 Hz, CH = CH), 7.37 (d, 2H, *J* = 8.5, ArH), 7.13–7.11 (m, 2H, ArH), 6.79 (d, 1H, *J* = 8.2, ArH), 6.50 (d, 1H, *J* = 15.9 Hz, CH = CH) ppm. ^13^C-NMR (75 MHz, DMSO-d_6_): *δ* = 215.8, 173.2, 165.2, 154.1, 149.5, 148.2, 146.0, 136.1, 129.0, 125.7, 123.6, 122.5, 116.2, 115.6, 112.8 ppm. HRMS (ESI-TOF) *m/z* Calcd for C_18_H_13_O_4_S_3_ [M + H]^+^: 388.9976; found: 388.9972.

### Various cinnamic acids derivatives

#### 2-(Methylsulfonylthio)ethyl 3,4-substitutedcinnamate (25–27) (general method)

**Scheme 4.**
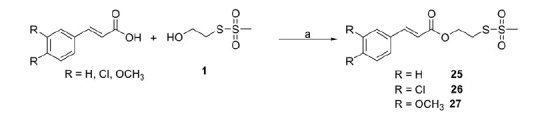
Reagents and conditions: (a) DCC, DMAP, anh. CH_2_Cl_2_ or THF, r.t., 1–4 h.

To a solution of *S*-2-hydroxyethyl methanesulfonothioate (**1**, 200 mg, 1.28 mmol) dissolved in anhydrous CH_2_Cl_2_ or THF (5 ml), cinnamic acid or (*E*)-3-(3,4-dichlorophenyl)acrylic acid, or (*E*)-3-(3,4-dimethoxyphenyl) acrylic acid (1.16 mmol), DCC (264 mg, 1.28 mmol), and DMAP (12 mg, 0.1 mmol) were added (Scheme 4). The reaction mixture was stirred for 1–4 h, at r.t. under inert atmosphere and it was monitored by TLC. After the completion of reaction, DCU was filtered and the solution was evaporated under reduced pressure. The obtained residue was taken up with iced EtOAc and the DCU filtered. The organic solution was washed firstly with a cold solution of 0.5 N HCl, then with a cold solution of 5% (w/w) NaHCO_3_ and finally with cold water and brine. The organic layer was dried with anhydrous Na_2_SO_4_, filtered, and evaporated to dryness to get a residue that was purified as indicated for each compound.

#### 2-(Methylsulfonylthio)ethyl cinnamate (25)

CC (silica gel; EtOAc/cyclohexane; 33:67). A yellow oil was obtained, which crystallised spontaneously in the fridge during the night. The solid was rinsed with ethyl ether to give white-cream crystals. Yield: 62%. Melting point 76.8–78.5 °C. ^1^H-NMR (300 MHz, DMSO-d_6_): *δ* = 7.73 (d, 1H, *J* = 15.9 Hz, CH = CH), 7.60–7.45 (m, 2H, ArH), 7.45–7.35 (m, 3H, ArH), 6.43 (d, 1H, *J* = 15.9 Hz, CH = CH), 4.52 (t, 2H, *J* = 6 Hz, CH_2_), 3.50 (t, 2H, *J* = 6 Hz, CH_2_), 3.39 (s, 3H, CH_3_) ppm. ^13^C-NMR (75 MHz, DMSO-d_6_): *δ* = 166.3, 145.7, 134.3, 131.0, 129.4, 128.9, 117.8, 62.8, 50.6, 34.8 ppm. HRMS (ESI) *m/z* Calcd for C_12_H_15_O_4_S_2_ [M + H]^+^: 287.04118; found: 287.04078.

#### (*E*)-2-(methylsulfonylthio)ethyl 3-(3,4-dichlorophenyl)acrylate (26)

Yellow oily residue that was purified by CC (silica gel; cyclohexane/EtOAc in gradient up to 65:35). The obtained residue was crystallised with EtOAc and the solid was rinsed with petroleum ether to give white crystals. Yield: 38%. Melting point 69.6–71.2 °C. ^1^H-NMR (300 MHz, CDCl_3_): *δ* = 7.67–7.55 (m, 2H, ArH and CH = CH), 7.48 (d, 1H, *J* = 8.2 Hz, ArH), 7.53 (dd, 1H, *J* = 1.6 and 8.2 Hz, ArH), 6.42 (d, 1H, *J* = 15.9 Hz, CH = CH), 4.52 (t, 2H, *J* = 6.3 Hz, CH_2_), 3.49 (t, 2H, *J* = 6.3 Hz, CH_2_), 3.39 (s, 3H, CH_3_) ppm. ^13^C-NMR (75 MHz, CDCl_3_): *δ* = 165.7, 143.1, 134.5, 134.1, 133.3, 130.9, 129.7, 127.1, 118.8, 62.6, 50.9, 35.0 ppm. HRMS (ESI) *m/z* Calcd for C_12_H_13_Cl_2_O_4_S_2_ [M + H]^+^: 354.96323; found: 354.96278.

#### (*E*)-2-((methylsulfonyl)thio)ethyl 3-(3,4-dimethoxyphenyl)acrylate (27)

Colorless oily residue, which was crystallised from diethyl ether into cream-white crystals. Yield: 45%. Melting point 99.8–102.7 °C. ^1^H-NMR (300 MHz, CDCl_3_): *δ* = 7.67 (d, 1H, *J* = 15.9 Hz, CH = CH), 7.12 (dd, 1H, *J =* 1.2 and 8.4 Hz, ArH), 7.05 (d, 1H, *J* = 1.2 Hz, ArH), 6.88 (dd, 1H, *J* = 8.4 Hz, ArH), 6.30 (d, 1H, *J* = 15.9 Hz, CH = CH), 4.51 (t, 2H, *J* = 6.3 Hz, CH_2_), 3.92 (s, 6H, OCH_3_), 3.50 (t, 2H, *J* = 6.3 Hz, CH_2_), 3.39 (s, 3H, CH_3_) ppm. ^13^C-NMR (75 MHz, CDCl_3_): *δ* = 166.5, 151.3, 149.1, 145.8, 126.9, 122.8, 114.5, 111.0, 109.7, 62.2, 55.9, 50.8, 35.1 ppm. HRMS (ESI) *m/z* Calcd for C_14_H_19_O_6_S_2_ [M + H]^+^: 347.06230; found: 347.06169.

### Rosmaricine derivatives

#### Extraction of rosmaricine (30)

**Scheme 5.**
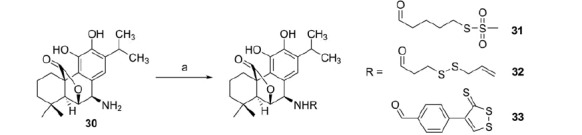
Reagents and conditions: (a) ROH (**6**, **9**, **11**), EDAC, HOBt, DIPEA, anh. DMF, r.t., 2–6 h.

A total of 150 g of rosemary dry leaves were divided into three 500 ml flasks and each portion was suspended in 200 ml of ethanol and kept in an ultrasound sonicator for 1 h (from 25 to 60 °C) (Scheme 5). After cooling, the mixture was filtered on a Buchner funnel and washed with ethanol. The residual leaves were treated twice with the same procedure. All the filtrates were gathered and concentrated at 100 ml of solvent volume and were treated following the procedure described by Boido et al.^53^ Yield (referred to dry leaves): 0.22%. Melting point 198.2–202.5 °C. ^1^H-NMR (DMSO-d_6_) *δ*: 8.05 (br s, 2H, collapsed with D_2_O); 6.77 (s, 1H); 4.37 (d, 1H, *J*= 2.47 Hz); 3.72 (d, 1H, *J*= 2.47 Hz); 3.30 (br s, 2H, collapsed with D_2_O); 3.26–3.09 (m, 2H); 1.84–1.70 (m, 1H); 1.60–1.18 (m, 5H); 1.11 (d, 3H, *J*= 6.88 Hz); 1.07 (d, 3H, *J*= 6.88 Hz); 0.94 (s, 3H); 0.77 (s, 3H). ^13^C-NMR (75 MHz, CD_3_OD): *δ* = 179.6, 144.2, 141.5, 136.2, 134.8, 127.4, 125.8, 118.2, 117.5, 79.7, 51.8, 37.9, 30.9, 30.4, 27.6, 27.5, 26.9, 22.0, 21.8, 18.9 ppm. HRMS (ESI) *m/z* Calcd for C_20_H_28_NO_4_ [M + H]^+^: 346.20183; found: 346.20127.

#### Amido derivatives of rosmaricine (31, 32, 33) (general method)

To a solution of the appropriate sulfurated acid (**6**, **9**, **11**; 0.29 mmol) in anhydrous *N,N-*dimethylformamide (1 ml) under argon and at 0 °C, HOBt (39.2 mg, 0.29 mmol), *N*,*N*-diisopropylethylamine (0.05 ml, 0.29 mmol), and EDAC (55.6 mg, 0.29 mmol) were added. After 5 min, rosmaricine (100 mg, 0.29 mmol) was added. The temperature was maintained at 0 °C with stirring for 2.5–6 h. After the completion of reaction, the solution was evaporated under reduced pressure. The obtained residue was taken up with ethyl acetate and washed firstly with a cold solution of 0.5 N HCl, then with a cold solution of 5% NaHCO_3_, finally with cold brine. The organic layer was dried with anhydrous Na_2_SO_4_, filtered and evaporated to dryness. The crude mixture was purified as indicated for each compound.

#### N-[5-(methylsulfonyl)thiopentanoyl]rosmaricine (31)

The green oil obtained was crystallised with a solution of diethyl ether, EtOAc and CH_2_Cl_2_ (in ratio 1:1:1) and rinsed with diethyl ether for several times to obtain a white solid. Yield: 38%. Melting point 121.1–124.2 °C (dec.). ^1^H-NMR (300 MHz, DMSO-d_6_): *δ* = 8.44 (br s, 2H, NH collapsed with D_2_O), 8.22 (br s, 2H, OH collapsed with D_2_O), 6.46 (s, 1H, ArH), 4.83 (d, 1H, *J* = 5.1 Hz, CH), 4.33 (s, 1H), 3.47 (s, 3H), 3.23–3.15 (m, 5H), 2.18–2.12 (m, 3H), 1.76–1.51 (m, 6H), 1.37–1.28 (m, 2H), 1.14–1.05 (m, 6H), 0.88 (s, 3H), 0.76 (s, 3H) ppm. ^13^C-NMR (75 MHz, acetone-d_6_): *δ* = 176.8, 171.5, 144.3, 141.6, 136.1, 126.6, 124.6, 118.6, 75.6, 50.7, 50.5, 49.8, 46.5, 38.1, 35.6, 34.9, 31.1, 30.9, 28.6, 27.5, 26.6, 24.5, 22.2, 21.98, 21.3, 18.9 ppm. HRMS (ESI): *m/z* Calcd for C_26_H_38_NO_7_S_2_ [M + H]^+^: 540.2090; found 540.2080.

#### N-[3-(allyldisulfanyl)propanoyl]rosmaricine (32)

The green oil was crystallised with diethyl ether and rinsed with the same solvent for three times to provide 15 mg of pure product. Moreover, the liquid filtrate was purified by CC (silica gel; CH_2_Cl_2_/MeOH_;_ in gradient up to 99.6:0.4). The fractions containing the purified product were gathered up to provide the final product as a light green-olive solid. Yield: 24%. Melting point 206.9–209.2 °C. ^1^H-NMR (300 MHz, acetone-d_6_): *δ* = 8.57 (br s, 1H, NH collapsed with D_2_O), 8.26 (br s, 2H, OH collapsed with D_2_O), 6.74 (s, 1H, ArH), 5.92–5.83 (m, 1H), 5.21–5.10 (m, 2H), 5.04 (t, 1H, *J* = 3.6 Hz), 4.57 (d, 1H, *J* = 3.6 Hz), 3.40 (d, 2H, *J* = 7.2 Hz), 3.33–3.10 (m, 3H), 3.03 (t, 2H, *J* = 6.3 Hz), 2.67 (t, 2H, *J* = 6.2 Hz), 2.08 (s, 1H), 2.03–1.87 (m, 1H), 1.57–1.43 (m, 3H), 1.22–1.14 (m, 6H), 1.00 (s, 3H), 0.88 (s, 3H) ppm. ^13^C-NMR (75 MHz, acetone-d_6_): *δ* = 176.8, 169.9, 144.3, 141.6, 136.0, 133.6, 126.4, 124.2, 118.7, 117.8, 75.6, 50.7, 50.6, 46.5, 41.5, 38.1, 35.1, 34.0, 31.1, 30.9, 27.5, 26.6, 22.2, 21.9, 21.3, 18.9 ppm. HRMS (ESI): *m/z* Calcd for C_26_H_36_NO_5_S_2_ [M + H]^+^: 506.2035; found 506.2026.

#### N-[4-(3-thioxo-3H-1,2-dithiol-4-yl)benzoyl]rosmaricine (33)

The crude residue was crystallised with a solution of ethyl acetate/diethyl ether (1:3). The resulting orange solid was purified by CC (silica gel; CH_2_Cl_2_/MeOH; in gradient up to 99:1). The fractions containing the purified product were rounded up and firstly crystallised with few drops of ethyl acetate and a solution of diethyl ether/petroleum ether (1:1). The solid was rinsed with diethyl ether/petroleum ether (1:1) to get orange crystals corresponding to the desired product. Yield: 19%. Melting point 243.2–249.7 °C. ^1^H-NMR (300 MHz, acetone-d_6_): *δ* = 9.12 (s, 1H), 8.04 (d, 2H, *J* = 7.4 Hz), 7.72 (d, 2H, *J* = 7.4 Hz), 6.82 (s, 1H), 5.28 (s, 1H), 4.72 (s, 1H), 3.50–3.20 (m, 2H), 1.70–1.35 (m, 4H), 1.35–1.05 (m, 8H), 0.99 (s, 3H), 0.92 (s, 3H) ppm. ^13^C-NMR (75 MHz, DMSO-d_6_): *δ* = 214.0 177.8, 166.2, 160.35, 156.1, 147.6, 145.2, 142.6, 136.8, 129.2, 128.2, 127.8, 127.5, 126.0, 124.5, 118.4, 76.3, 60.2, 50.9, 49.9, 46.6, 38.2, 31.4, 26.6, 2.1, 22.8, 21.9, 19.2 ppm. HRMS (ESI): *m/z* Calcd for C_30_H_32_NO_5_S_3_ [M + H]^+^: 582.1443; found 582.1447.

### STAT AlphaScreen-based assay

STAT3 inhibitory activity of the described compounds was tested by the AlphaScreen-based assay to evaluate the potential inhibition of the interaction between STAT3-SH2 domain and pTyr-containing peptides according to the previously reported procedure^54^. For the most interesting compounds, selectivity tests versus STAT1 were performed.

### Luciferase reported promoter activities assay

To measure the NF-κB promoter activity, HCT-116 cells were seeded in 100 mm dish and transiently transfected with 6 μg of DNA (NF-κB plasmid, containing luciferase reporter gene^57^ with turbofect reagent (Carlo Erba Reagents). After 24 h, cells were seeded in 48-well plates (40.000 cells per well). On the next day, cells were incubated with our compounds for 2 h (pre-treatment) and then TNF-α was added in every well at (10 ng/ml). After 5–6 h, luciferase activity was measured by using Neolite reagent (PerkinElmer Life Sciences, Waltham, MA) according to the manufacturer’s instructions^58^.

### STAT3 luciferase reporter gene assay

STAT3 reporter HeLa stable cell lines (Signosis Inc, Santa Clara, CA) were incubated in a 96-well microplate for 24 h. Cells were pretreated with test compounds for 2 h, and 10 ng/ml (w/v) of oncostatin M were applied and incubated for 4 h. Cells were washed with medium not supplemented with phenol red, and Steady-Glo^®^ reagent (Promega, Madison, WI) was applied. After 15 min incubation, the signals were detected by ARVO Light 1420 (PerkinElmer Life Sciences, Waltham, MA). The relative signal intensity was calculated in each well as the ratio for the mean signal of vehicle.

### MTT-assay

Following the same protocol used for previous cytotoxicity assays by N. Ferri^55^, cells were seeded in 48-well plate (40.000 cells/well) and, after 24 h, they were incubated with different concentrations of our compounds for 48 h.

The determination of the conversion of MTT (3-(4,5-dimethyl-2-thiazolyl)-2,5-diphenyl-2H-tetrazolium bromide) to formazan was determined by using a commercially available kit (Millipore, Billerica, MA), according to the manufacturer’s instructions.

## Results and discussion

### Synthesis

Six sulfurated moieties were previously described, and were prepared accordingly: **1** ^59^, **3** ^60^, **5** ^61^, **6** ^62^, **9** ^63^, **11** ^63^. The alcohols **2** and **4** were synthesised according to Scheme 1.

The methyl esters **7** and **10** were obtained in good yields, through the condensation of the carboxylic acids **6** and **9** with methanol, using DCC and DMAP as coupling agents. Finally, the sulfone **8** was prepared by reacting sodium methanesulfinate with 5-bromovaleric acid (Scheme 1).

Ferulic acid hybrids have been obtained by coupling the MEM-protected acid (**35**) with the proper alcohols (S-2-hydroxyethyl methanesulfonothioate (**1**), S-4-hydroxybutyl methanesulfonothioate (**2**), 2-(allyldisulfanyl)ethanol (**4**)), with the S-2-aminoethyl methanesulfonothioate (**3**) and with 5-(4-hydroxyphenyl)-1,2-dithiol-3-thione (**5**) to achieve the intermediates **36**–**40**. After deprotection with trifluoroacetic acid (TFA) at r.t., the final compounds (**14**–**18**) were obtained (Scheme 2).

Caffeic acid hybrids were obtained through a similar synthetic route. However, in this case, the protection of the catechol group was better achieved by treating caffeic acid methyl ester (**20**) with NaH and then with MEM-Cl. After 5 N NaOH hydrolysis, the obtained protected acid (**42**) was coupled with the sulfurated moieties **1** and **3–5** to achieve the intermediates **28**, **29**, **43** and **44** and finally, the treatment with TFA at r.t. gave the expected hybrids **21**–**24** (Scheme 3).

**Figure 4.**
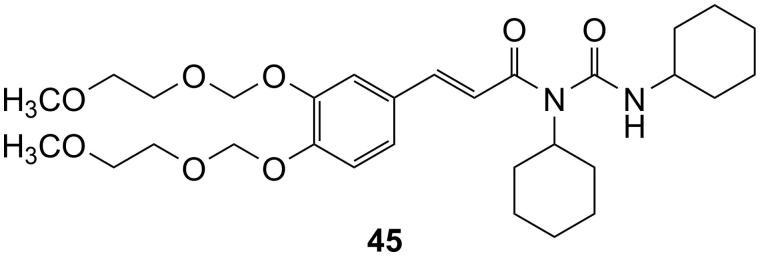
Structure of compound **45**.

To be noted that, during the coupling reaction between compound **42** and 2-(allyldisulfanyl)ethanol (**4**) and in a lower extent with **1**, the formation of the N-acylurea (**45**) of the acid was observed (Figure 4).

Compounds **25**–**27** were synthesised by coupling the S-2-hydroxyethyl methanesulfonothioate (**1**) with cinnamic acid, 3,4-dichlorocinnamic and 3,4-dimethoxycinnamic acid, respectively (Scheme 4).

Finally, rosmaricine (**30**) was acylated with the sulfurated acids **6**, **9**, and **11**, in presence of EDAC and HOBt as coupling reagents, according to Scheme 5.

### Biological results

The new sulfurated-drug hybrids, together with their parent compounds have been submitted to the AlphaScreen-based assay, to investigate their ability to bind STAT3 directly, through the evaluation of the inhibition of the binding of SH2-containing proteins to their correspondent phosphopeptides, the physiological ligands. Moreover, in order to check the selectivity of action of our molecules on STAT3, their ability to interact with SH2-domain of STAT1, a family member protein exhibiting a high degree of sequence homology to STAT3 and tumor suppressive properties in many systems, has also been tested. Results, expressed as % of protein inhibition at 30 μM or as IC_50_ (μM), are reported in Tables 1 and 2, together with the cytotoxic activity on HCT-116 cell line (MTT assay) expressed as IC_50_ (μM). Since AlphaScreen is a cell-free assay, not always there is a correspondence between the potency of STAT3 inhibition and cytotoxicity, probably due to not optimal physicochemical properties of the tested compounds, such as solubility and chemical stability in the culture medium and cell permeation. For this reason, compounds which exhibited antiproliferative activity, were also evaluated for their ability to inhibit STAT3 reporter activity on cells (HeLa). Results, expressed as IC_50_ (μM), are reported in Tables 1 and 2. In addition, the ability of the most active compounds to inhibit the promoter activity of NF-κB on HCT-116 cells was also investigated (Luciferase assay) and it is reported as IC_50_ (μM) in the same tables.

**Table 1.** Biological activities of sulfurated parent compounds (**1**–**6**, **9**, and **11**) and some related compounds (**7**, **8**, and **10**).

	AlphaScreen Assay				STAT3 reporter assay	NF-κB promoter assay	Cytotoxicity
Compd	STAT3 % inhibition ± SD at 30 μM	STAT3 IC_50_ ±SD (μM)	STAT1 % inhibition ± SD at 30 μM	STAT1 IC_50_ ±SD (μM)	(HeLa) IC_50_±SD (μM)	(HCT-116) IC_50_ ±SD (μM)	(HCT-116) IC_50_ ±SD (μM)
**1**	11.6^a^	>30	n.t.	n.t.	n.t	NA	NA
**2**	88.4 ± 0.5	4.4 ± 0.3	73.7 ± 1.4	8.6 ± 0.7	n.t	n.t.	NA
**3**	−10.2^b^	>30	n.t.	>3^c^	n.t	n.t.	NA
**4**	55.7 ± 2.5	22.2 ± 3.0	11.7 ± 6.0	>30	n.t	n.t.	NA
**5**	43.7^a^	>30	n.t.	n.t.	n.t	36.7 ± 11.4	71.2 ± 15.0
**6**	72.5 ± 4.8	4.7 ± 1.1	n.t.	>3^d^	n.t.	NA	NA
**7**	99.6 ± 0.1	1.9 ± 0.2	70.8 ± 1.2	20.1 ± 0.4	n.t.	n.t.	NA
**8**	3.8 ± 2.8	>30	n.t.	n.t.	n.t.	n.t.	NA
**9**	43.9 ± 3.6	>30	n.t.	n.t.	n.t.	n.t.	NA
**10**	42.0 ± 4.7	>30	11.2	>30	n.t.	n.t.	NA
**11**	−1.2 ± 6.0	>100	n.t.	n.t.	>100	NA	NA

n.t.= not tested.

NA = not active up to 100 μM.

a Mean of two experiment.

b Inhibition at 3 μM: 46.8 ± 10.1%.

c Inhibition at 3 μM: 14.5 ± 7.1%.

d Inhibition at 3 μM: 8.6 ± 1.7%.

**Table 2.** Biological activities of parent compounds (ferulic acid **12**, caffeic acid **19**, and rosmaricine) and their sulfurated derivatives.

	AlphaScreen Assay				STAT3 reporter assay	NF-κB promoter assay	Cytotoxicity
Compd	STAT3 % inhibition ± SD at 30 μM	STAT3 IC_50_±SD (μM)	STAT1 % inhibition ± SD at 30 μM	STAT1 IC_50_ ±SD (μM)	(HeLa) IC_50_ ±SD (μM)	(HCT-116) IC_50_ ±SD (μM)	(HCT-116) IC_50_ ±SD (μM)
**12**	0.2 ± 8.2	>30	n.t.	n.t.	n.t.	NA	NA
**13**	2.1 ± 2.3	>30	n.t.	n.t.	n.t.	n.t.	NA
**14**	110.1 ± 0.2	0.9 ± 0.0	110.5 ± 0.6	7.0 ± 0.7	65.4 ± 1.0	116.3 ± 2.9	50.4 ± 5.9
**15**	100.0 ± 0.1	0.3 ± 0.0	86.0 ± 6.7	1.8 ± 0.4	57.5 ± 1.1	94.0 ± 4.6	64.4 ± 9.1
**16**	107.8 ± 0.1	1.8 ± 0.5	4.1 ± 5.4	>30	n.t	n.t	NA
**17**	67.9 ± 0.8	7.8 ± 0.6	12.0 ± 4.7	>30	n.t	n.t.	NA
**18**	51.3 ± 0.7	28.5 ± 0.7	44.2 ± 1.9	>30	n.t	NA	NA
**19**	11.0 ± 5.4^a^	>30	n.t.	n.t.	n.t	NA	NA
**20**	12.8 ± 1.1	>30	n.t.	n.t.	n.t	n.t.	90.8^b^
**21**	100.1 ± 0.1	0.8 ± 0.2	85.7 ± 4.3	17.0 ± 1.2	60.6 ± 2.8	127.8 ± 8.9	123.2 ± 17.3
**22**	99.8 ± 0.1	0.9 ± 0.1	−17.8 ± 0.3	>30	n.t.	n.t.	NA
**23**	70.0 ± 1.7	5.5 ± 0.2	37.7 ± 7.5	>30	94.4 ± 26.1	163.3 ± 11.1	73.0 ± 8.0
**24**	66.7 ± 0.5	13.2 ± 0.9	21.5 ± 1.8	>30	40.0 ± 1.5	40.6 ± 3.0	46.7 ± 4.1
**25**	100.0 ± 0.0	0.5 ± 0.0	100.3 ± 0.2	3.6 ± 0.4	72.5 ± 4.4	NA	95.2 ± 8.9
**26**	100.0 ± 0.1	0.3 ± 0.0	103.8 ± 1.7	1.9 ± 0.2	n.t	n.t.	NA
**27**	99.7 ± 0.1	0.3 ± 0.0	68.9 ± 10.2	20.9 ± 4.9	n.t	n.t.	NA
**28**	100.0 ± 0.1	0.6 ± 0.1	98.4 ± 0.9	6.3 ± 0.5	n.t	n.t.	NA
**29**	100.1 ± 0.1	1.1 ± 0.0	63.3 ± 0.5	19.5 ± 0.3	n.t	n.t.	n.t.
**30**	99.7 ± 0.0	1.9 ± 0.1	91.8^b^	10.0 ± 0.6	42.4 ± 2.4	43.4 ± 6.9	65.2 ± 2.3
**31**	100.1 ± 0.1	0.2 ± 0.0	99.9 ± 0.3	2.8 ± 0.1	>100	53.3 ± 10.9	73.8 ± 0.9
**32**	99.5 ± 0.0	2.3 ± 0.4	23.9 ± 0.4	>30	37.0 ± 0.8	13.0 ± 0.7	3.5 ± 2.8
**33**	94.5 ± 0.4	2.9 ± 0.2	58.8 ± 5.9	19.7 ± 4.0	>100	73.0 ± 13.2	65.4 ± 3.9

n.t.= not tested.

NA = not active up to 100 μM.

a Inhibition at 300 μM: 61.8 ± 2.5%.

b Mean of two experiments.

First of all, it is observed that among the sulfurated moieties, that were used to form the conjugated molecules, only three of them (**2**, **4,** and **6**) exhibited IC_50_ < 30 μM for the binding to STAT3-SH2 domain (AlphaScreen assay) (Table 1). Particularly, (4-methanesulfonylthio)butanol (**2**) was more active than the lower homolog 2-(methanesulfonylthio)ethanol (**1**), as the methyl ester **7** exhibited a higher activity than the corresponding free acid **6**. Thus, the binding activity seems somewhat increasing with the increasing lipophilicity of the molecule; this observation holds also for the feruloyl esters **15** and **14** of the two homologous alcohols **2** and **1**, respectively. However, no difference was observed between ester **10** in comparison to free acid **9**.

The replacement of the thiosulfonate function in compound **6** (IC_50_= 4.7 μM) with a sulfone group (compound **8**) led to the loss of activity on STAT3. Thus, the methanethiosulfonate moiety is confirmed as crucial for the inhibitory activity, probably through the interaction with the thiol group of cysteines in STAT3.

The same set of compounds did not display any cytotoxicity versus the HCT-116 cancer cells up to 100 μM concentration, with the only exception of 4-hydroxyphenyldithiolethione (**5**) (IC_50_= 71.2 μM).

Also, the phenolic parent compounds, ferulic (**12**) and caffeic (**19**) acids and their simple methyl esters (**13** and **20**) did not bind, at least up to 30 μM concentration, to STAT3-SH2 domain (Table 2), confirming that they are not "direct" STAT3 inhibitors and that they may act on other steps of STAT3 pathway.

Very interestingly, all the sulfurated hybrid molecules derived from ferulic acid (**14**–**18**), caffeic acid (**21**–**24**) and from other cinnamic acids (**25**–**29**) (rosmaricine derivatives will be discussed later) were able to strongly bind STAT3-SH2 domain. Some of them were also able to inibit the NF-κB transcriptional activity in HCT-116 cells and STAT3 reporter activity in HeLa cells. Accordingly, six hybrid compounds inhibited HCT-116 cell proliferation *in vitro*, with IC_50_ in the range 47–123 μM. The inhibition of both transcription factors exerted by esters **14**, **15**, **21**, **23**, and **24** in cell assays and their antiproliferative activity versus HCT-116 cells indicate that the ester function may survive to the action of cellular esterases and to the deactivating power of GSH that is commonly overexpressed in cancer cells. In particular, the hybrid molecules containing the thiosulfonate function exhibited IC_50_ in the range 0.3–1.8 μM. These compounds were also rather selective STAT3 inhibitors in comparison to STAT1, in spite of the high degree (78%) of sequence homology that characterises the two proteins, and exhibited a higher selectivity index (S.I.= IC_50_ STAT3/IC_50_ STAT1: range 6–33, plus an outranging value of ˜70) in comparison to the previously described^42^ ^,^ ^43^ thiosulfonate hybrid **a–i** (S.I: in the range 2–12, plus an outranging value of 43).

The IC_50_ values for the inhibition of STAT3 and STAT1 of the thiosulfonate subset of compounds (but also for other subsets) did not run parallel, indicating that the structural and physicochemical requirements to hit the two proteins are rather different; particularly it is observed that the two potent STAT3 inhibitors **15** and **26** were only moderately selective (S.I. = 6 and 6.3, respectively), but the other potent STAT3 inhibitor **27** was 10-fold more selective (S.I.= 69.7).

Although, the real mechanism has not been elucidated yet, it is possible that methanethiosulfonates covalently modify STAT3 cysteines and that these structural changes lead to altered STAT3 signaling. Such kind of STAT3 inhibition would be challenging because healthy cells could also be affected. However, as the above hybrid molecules were able to discriminate between the two STAT proteins, they might also display an acceptable selectivity versus the cysteine-containing proteins of the healthy cells.

The activities of the unsubstituted cinnamic ester (**25**) and the non-phenolic 3,4-disubstituted cinnamic esters (**26**–**28**) and amide (**29**) are comparable to those of the ferulic and caffeic esters and amides (**14–16, 21, 22**), suggesting that the phenolic function (and the related anti-oxidant activity) is not essential for a valid inhibitory activity on STAT proteins, while it might profitably address the molecules to other relevant targets, eventually concurring to the antiproliferative activity (as for compounds **23** and **24**).

Moreover, it is not excluded that some contribution to the binding capability, mostly related to the sulfurated moieties, could derive from the Michael addition propensity of the carbonyl-conjugated double bond^66^.

The STAT3 inhibitory activities of the variously substituted cinnamic esters were confined within a rather narrow range (IC_50_ from 0.3 μM to 0.9 μM), that includes also the IC_50_ value (0.7 μM) of the corresponding ester of the non-aromatic valproic acid (**a**) previously described. Therefore, a further extension of the present exploratory study to other esters of aromatic and aliphatic acids with methanesulfonylthio-ethanol and –butanol is worthy to be pursued.

The simple esterification of ferulic and caffeic acids with methanol, while surely increasing the molecular lipophilicity, did not apparently improved their poor STAT3 inhibitory activity; nevertheless, some increase of cytotoxicity was observed in the case of caffeic acid methyl ester **20** (IC_50_=90.8 μM), in line with the previously observed increase of cytotoxicity in the phenylethyl and phenylpropyl esters of caffeic acid^28^.

As far as allyldisulfide derivatives, they seem to interact in a weaker way than methanethiosulfonates with the STAT3-SH2 domain. Nevertheless, the caffeic acid derivative **23**, besides STAT3 inhibition (IC_50_ = 5.5 μM), showed also moderate NF-κB inhibitory activity and antiproliferative activity on HCT-116 cells (IC_50_=73.0 μM); thus, exhibiting interesting properties to be further investigated for a potential multi-target anticancer activity.

Dithiolethione drug hybrids **18** and **24** were the weakest and less selective inhibitors on STAT3-SH2 domain compared to the corresponding methanethiosulfonate and allyldisulfide derivatives in the AlphaScreen-based assay; however, the caffeic acid derivative **24**, exhibited a very good profile, inhibiting both the transcription factors (STAT3 AlphaScreen: IC_50_ = 13.2 μM; STAT3 reporter assay: IC_50_ = 40.0 μM and NF-κB promoter activity: IC_50_ = 40.6 μM) and the HCT-116 cell proliferation (IC_50_ = 46.73 μM) at comparable concentrations.

Of particular interest were the results of the testing of rosmaricine (**30**) and of its amidic derivatives (**31–33**) of sulfurated acids, namely 5-(methanesulfonylthio)pentanoic acid (**6**), 3-(allyldisulfanyl)propionic acid (**9**), and 4-(3-thioxo-3H-1,2-dithiol-4-yl)benzoic acid (**11**).

First of all, it is observed that rosmaricine itself was able to strongly bind to the STAT3-SH2 domain (IC_50_ = 1.9 μM) and to inhibit STAT3 reporter activity in HeLa cells, as well as NF-κB transcriptional activity in HCT-116 cells; thus, exerting a moderate antiproliferative activity (IC_50_ = 65 μM) against the same cancer cells.

The amidification of rosmaricine with the methanesulfonylthiopentanoic acid (**31**) produced a 10-fold increase of inhibitory potency on STAT3 (IC_50_ = 0.2 μM), while the linkage with the allyldisulfanyl and the dithiolethione moieties left unchanged or even slightly reduced the rosmaricine activity on STAT3. Thus, once more, the thiosulfonate moiety resulted the most potent enhacer for the binding to STAT3-SH2 domain, while the other relevant parameters were affected in different directions and degree.

Indeed, the hybrids **31** and **33**, despite the different IC_50_s versus STAT3 (0.2 and 2,9 μM, respectively), exhibited a quite reduced STAT3 reporter activity, but unchanged activity versus NF-κB and unchanged cytotoxicity in comparison to the parent rosmaricine. On the contrary, the 3-(allyldisulfanyl)propanoyl derivative **32** exhibited an unchanged STAT3 reporter activity, in comparison to rosmaricine, but an increased potency versus the NF-κB transcription factor and a 19-fold increase of antiproliferative activity (IC_50_ = 3.5 μM). Versus the normal human smooth muscle cells (hSMC), compound **32** exhibited an IC_50_ = 21.2 μM, with a selectivity index of 6. The valuable cytotoxicity of **32** on HCT-116 cells, was confirmed, even at lower level against MCF-7 (hormone sensitive breast cancer) cells.

Soon after its first isolation, rosmaricine was investigated for anticancer activity by Russian authors, but results were not illustrated in the only available scanty Chemical Abstracts report^67^. On the other hand, rosmaricine and some derivatives^53^, where the cathecol groups were either free or methylated resulted inactive or endowed with borderline activity against the lymphocytic leukemia P388, when injected i.p. in mice, 24 h after the tumor implants (data from National Cancer Institute, Bethesda, MD).

More recently, by virtual screening and docking studies of thousands of compounds^68^, rosmaricine (as well as emetine and two esters of caffeic and ferulic acids) was suggested as potential strong inhibitors of epidermal growth factor receptor (EGFR). Indeed, some natural molecules closely related to rosmaricine, as carnosic acid, carnosol, and rosmanol, have been shown to possess antiproliferative activity on a variety of cancer cell lines, and their activity is mainly related to generation of ROS and inactivation of STAT3^48–50^.

Thus, the same factors may explain the present observed antiproliferative activity on HCT-116 cells of rosmaricine and its derivatives **31** and **33**, while the outstanding cytotoxicity of **32** might be related to the additional interaction of its allyldisulfanyl moiety with NF-κB moiety and/or other targets.

All these data underline the interesting activity profiles of rosmaricine and its derivatives (in particular compound **32**), which are worthy of further investigation as potential multi-target antiproliferative agents.

## Conclusions

Starting from some cinnamic acids (particularly ferulic and caffeic) and the diterpenoid rosmaricine, 17 new sulfurated hybrid molecules were synthesised and their ability to *in vitro* inhibit STAT3 and NF-κB transcription factors as well as their cytotoxicity on the human colon carcinoma cell line (HCT-116) were evaluated.

All resulted hybrid compounds were able to directly and strongly inhibit STAT3 transcription factor (AlphaScreen assay) and most of them were also shown to inhibit NF-κB transcriptional activity in HCT-116 cells and the proliferation of the said cancer cells. Thus, once more, the conjugation of molecules, each endowed with even weak affinity versus STAT3 and/or NF-κB transcription factors, produced strong inhibitors of the latter factors, which (besides the other peculiarities of each moiety) gave rise to valuable antiproliferative activity on HCT-116 cancer cells.

STAT3 was more potently inhibited in comparison to STAT1, in spite of the high sequence homology between the two proteins, indicating the possibility to discriminate among thiol containing proteins and eventually to achieve novel hybrid compounds able to strongly hit the STAT3 factor with only modest involvement of healthy cell proteins.

In particular, the methanethiosulfonate drug hybrids interact with STAT3-SH2 domain more potently than the corresponding allyldisulfide and dithiolethione derivatives.

The parent compounds were completely devoid of inhibitory activity at the tested concentrations, or, in a few cases (**2**, **4,** and **6**) their potency was quite lower than that of the hybrids, excluding the observed activity of the latter could be related to the hydrolytic liberation of the former.

In spite of the very good STAT3 inhibition resulting from the AlphaScreen assay, only nine compounds exhibited cytotoxicity on colon cancer cells and inhibition of STAT3 reporter gene activity, probably due to unsuitable physicochemical properties of some of the tested compounds, which need to be further investigated and optimised.

Eight hybrids (**14**, **15**, **21**, **23**–**25**, **31**, and **33**) and rosmaricine (**30**) exhibited moderate cytotoxicity, with IC_50_ in the range 46.7–123.2 μM, but the hybrid **32** (N-(3-allyldisulfanyl)propanoyl-rosmaricine) displayed a valuable cytotoxicity (IC_50_= 3.5 μM), that was confirmed at lower level, in MCT-7 breast cancer cells.

On the whole, the most interesting compounds were the two thiosulfonate-ferulic acid hybrids (**14** and **15**), the allyldisulfide-caffeic acid hybrid (**23**), the dithiolethione-caffeic acid hybrid (**24**), and the foresaid rosmaricine hybrid (**32**), besides rosmaricine (**30**) itself. All of them inhibited both STAT3 and NF-κB transcription factors and exhibited from moderate to good antiproliferative activity and may represent hit compounds for developing multi-target anticancer agents.
